# Sustainable Production of High-Performance Antimicrobial Scaffold via an Engineered *Halomonas* Dual-Product Factory

**DOI:** 10.3390/biom16060889

**Published:** 2026-06-17

**Authors:** Ehab Marwan-Abdelbaset, Xiaoyun Lu, Dan Tan

**Affiliations:** 1 Key Laboratory of Biomedical Information Engineering of Ministry of Education, School of Life Science and Technology, Xi’an Jiaotong University, Xi’an 710049, China; ehab_emm@azhar.edu.eg (E.M.-A.); luxy05@xjtu.edu.cn (X.L.); 2 Department of Botany and Microbiology, Faculty of Science, Al-Azhar University, Nasr City, Cairo 11884, Egypt

**Keywords:** metabolic engineering, *Halomonas bluephagenesis* TD01 as (NGIB), one-step fermentation, co-production, hyaluronic acid, PHB-nanoparticles, antimicrobial scaffold

## Abstract

This study presents a transformative “one-pot” biorefinery approach for the simultaneous production of hyaluronic acid (HA) and polyhydroxybutyrate (PHB) using an engineered, non-pathogenic *Halomonas bluephagenesis* TD01 chassis. By leveraging the principles of Next-Generation Industrial Biotechnology (NGIB), a one-step fermentation process was developed in nutrient-rich 40-LBG-Y medium, achieving a balanced metabolic flux that yielded 1.99 g/L and high-molecular-weight (HMw) HA (9.6 × 10^6^ Da) as the highest HA-Mw reported by heterogeneous bacteria, alongside intracellular PHB (0.68 to 1.6 g/L). A bioactive HA-PHB nanoparticle scaffold was fabricated, exhibiting a highly porous, interconnected 3D sponge-like architecture with a significant particle size shift from 12 nm to 450 nm, confirming successful polymer complexation. Antimicrobial evaluations revealed that the scaffold exhibited preliminary antimicrobial potential against representative Gram-positive and Gram-negative strains against *Staphylococcus aureus*, *Klebsiella variicola*, and *Candida albicans*. Notably, while *Pseudomonas aeruginosa* metabolically exploited purified HA, the integrated scaffold reversed this effect, providing preliminary antimicrobial potential by sterically hindering bacterial hyaluronidases. Furthermore, *Halomonas*-derived HA consistently outperformed Moringa oil and complex emulsions in preliminary tests against a wide range of pathogenic microbes. These results demonstrate that this dual-product platform provides a sustainable, cost-effective source of high-performance functional materials for advanced antimicrobial coatings and clinical wound management.

## 1. Introduction

One-step fermentation for the simultaneous production of hyaluronic acid (HA) and polyhydroxybutyrate (PHB) represents a promising “one-pot” biorefinery approach, combining the synthesis of a high-value extracellular polysaccharide (HA) with an intracellular biodegradable plastic (PHB) [1,2]. Historically, HA and PHB were synthesized by separate hosts (such as *Streptococcus* species like *S. zooepidemicus* for HA, and *Cupriavidus*, *Halomonas*, or *Bacillus* species for PHB); however, advanced fermentation now enables their combined production [3]. Additionally, traditional commercial HA production relies on pathogenic *Streptococcus* hosts, necessitating rigorous and costly purification steps to remove endotoxins and exotoxins. As was reported with *S. zooepidemicus*, the highest HA production achieved was 2.825 g using a L/4.5 L bioreactor with controlled pH (8.0) and medium containing molasses [4]. In another study, a mutant *S. zooepidemicus* strain in a semi-continuous fermentation process consisting of two-stage 3L bioreactors was developed, in which 1.01 g/L/h productivity of hyaluronic acid was obtained, and after recombinant hyaluronidase SzHYal was added into the second stage bioreactor at 6 h, 14.60 g/L, and 29.38 g/L, respectively [5].

**Figure 1:**
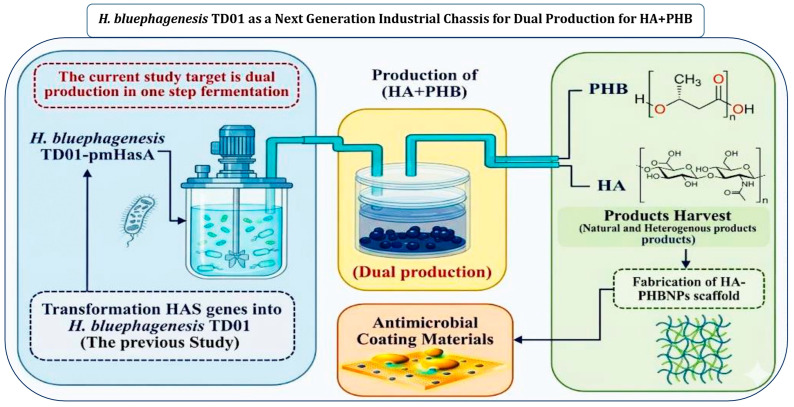
Schematic overview of the engineered *H. bluephagenesis* TD01 platform for the cost-effective production of HA and PHB. The diagram illustrates the integrated workflow of next-generation industrial biotechnology (NGIB), funneling into a one-step fermentation process for dual-product harvesting. The final output of this platform is the synthesis of endogenous and heterogeneous metabolites (PHB and HA), which are processed into HA-PHB nanoparticle scaffolds for use as antimicrobial coating materials [8].

Economically viable biopolymer production requires an optimal microbial chassis like *Halomonas bluephagenesis* TD01. As a robust, non-pathogenic extremophile, this strain enables the open, unsterile, and continuous fermentation central to Next-Generation Industrial Biotechnology (NGIB) [6,7,8]. By integrating the *pmHasA* gene, *H. bluephagenesis* excels as a dual-product factory, converting glucose into both PHB and high-value hyaluronic acid (HA) within a single bioreactor batch [8,9,10]. As illustrated in Figure 1, this one-step co-production strategy offers distinct industrial advantages over traditional methods: Utilizing a non-pathogenic host eliminates the costly, rigorous purification steps required to remove streptococcal endotoxins and exotoxins [11]; merging two polymer pathways into one batch eliminates separate fermentation stages, drastically reducing energy, time, and operational costs [11,12]; minimizing overall processing requirements significantly enhances industrial throughput.

Hyaluronic acid (HA) biosynthesis is governed by a deeply complex metabolic network that connects glycolysis, nucleotide sugar metabolism, and plasma membrane dynamics. This interconnected system dictates both the quantity of HA produced and the molecular weight (MW) of the polymer, which controls its biological activity [13,14]. The biosynthetic pathway consumes vast amounts of cellular carbon and energy, directly altering the global metabolic state of the cell. The heavy consumption of precursor sugars pulls carbon and ATP away from baseline cellular respiration. In many cell types, up-regulating HA synthesis can accelerate the flux of glucose through the HBP and entirely shift cellular energy profiles (e.g., driving shifts between glycolysis and mitochondrial respiration) [15].

PHB biosynthesis is an intricate anabolic process where microbes convert carbon sources into intracellular polyester granules. This pathway acts as a crucial metabolic sink under stress and is driven by the dynamic flux of central carbon metabolism and tightly regulated enzyme kinetics [16]. The primary pathway utilizes three sequential enzymatic steps to convert acetyl-CoA into PHB. PHB is an overflow metabolite and an electron sink. Biosynthesis is strongly promoted during conditions of nutrient imbalance (e.g., limitation of nitrogen, phosphorus, or oxygen) combined with an excess carbon supply [17].

The rise of global antimicrobial resistance (AMR) has compromised conventional antibiotics, driving the urgent need for innovative materials that maximize antibacterial efficiency while minimizing cellular toxicity and further resistance [18].

The biological effects of HA are highly molecular weight (MW)-dependent: high-MW variants (>5 × 10^5^ Da) serve as tissue fillers and immunosuppressants, whereas low-MW oligomers (6 × 10^3^ to 10^4^ Da) promote angiogenesis and immunostimulants [19,20]. These diverse properties drive HA’s widespread use in both indigenous and modern clinical formulations to accelerate wound closure and treat resistant skin conditions [21]. Additionally, HA provides concentration- and MW-dependent antimicrobial protection [22]. For instance, it successfully prevents chronic urinary tract infections by replacing the damaged GAG layer of the bladder wall [23]. While HA lacks efficacy against E. coli, it exhibits robust, dose-dependent growth inhibition against 15 standard ATCC strains, multiple *Pseudomonas* species, and prominent clinical pathogens, including *Streptococcus mutans*, *Enterococci*, *Staphylococci*, and *Candida* [22,24].

The clinical utility of pure hyaluronic acid (HA) is limited by rapid hydrolysis from bacterial hyaluronidases and poor mechanical stability in aqueous physiological environments due to its high hydrophilicity [25]. To address these drawbacks, researchers have developed chemically crosslinked HA composites, nanogels, and nanoparticles. These engineered systems extend the biological half-life of HA while establishing a stable matrix for sustained drug delivery [26,27].

Polyhydroxyalkanoates (PHAs) are sustainable, renewable polyesters whose high tensile strength, flexibility, and excellent biocompatibility make them ideal for three-dimensional (3D) tissue engineering scaffolds [28,29]. Within this class, poly(3-hydroxybutyrate) (PHB) is highly promising for nerve and tissue regeneration [30,31]. As a nanoparticle formulation, PHB provides a microenvironmentally responsive, high-capacity drug-loading matrix and a strong, slowly degrading mechanical “skeleton” for long-term tissue support [32,33]. However, its inherent hydrophobicity and lack of cell-recognition sites naturally limit cell attachment [34,35]. Integrating highly hydrophilic HA directly overcomes these limitations, drastically improving the composite’s wetting capabilities to promote rapid infiltration by blood, proteins, and cells [36,37,38].

Moreover, this blend effectively compensates for HA’s natural mechanical weakness and rapid enzymatic degradation. By anchoring HA within the stable PHB matrix, the scaffold survives long enough for tissue repair while leveraging HA’s natural bioactivity [39,40,41]. This allows the composite material to actively bind to cellular receptors (such as CD44 and RHAMM), transforming an otherwise inert physical structure into a dynamic environment that signals cells to migrate, proliferate, and differentiate. Scaffolds serve as essential biomaterials that promote cell-biomaterial interaction, allow the passage of nutrients and gases, and biodegrade at a rate synchronized with tissue regeneration [42,43,44].

While HA and PHB hydrogel scaffolds have been individually recognized for their biocompatibility, their combined application as a dedicated antimicrobial scaffold for treating bacterial infections remains largely unexplored. However, existing literature confirms that these polymers possess significant anti-inflammatory and antioxidant properties, which are essential for profound wound healing [45,46,47]. Polymer-based delivery systems are widely documented for their ability to enhance the bioavailability of therapeutic agents and provide controlled drug-release kinetics. Given the distinct medicinal properties of HA and PHB, researchers have previously employed HA and PHB nanoparticles (NPs) either independently or in combination with other chemical agents to validate polymer-based delivery efficacy [48,49]. In this study, the HA-PHB composite was utilized as a standardized scaffold model.

Simultaneously, the incorporation of Moringa oil introduces potent antioxidant, anti-inflammatory, and antimicrobial properties [50,51]. This ensures that as HA signals new cells to grow and differentiate, the Moringa oil actively protects the newly forming tissue from infection and oxidative stress, creating an optimized microenvironment for successful healing. In addition to synthetic biopolymers, natural extracts like Moringa oil (from Moringa oleifera) have gained attention for their high antimicrobial activity and oxidative stability [52,53]. However, the volatility and sensitivity of Moringa oil to light and air limit its long-term efficacy [54]. Embedding Moringa oil within a hyaluronic acid polymer matrix or emulsion may potentially increase its stability and prolong its action. This study investigates whether such complexation provides a synergistic boost to the intrinsic anti-microbial properties of *Halomonas*-derived HA.

The primary objective of this research is to evaluate the metabolic relationship and carbon flux between HA biosynthesis and PHB accumulation within the engineered *H. bluephagenesis* TD01 chassis during a simplified, one-step fermentation process. The second objective is to fabricate and characterize a bioactive HA-PHB nanoparticle scaffold that leverages the structural stability of PHB and the biological activity of HA to form a cohesive 3D network, overcoming the limitations of HA and PHB separately in medical uses through scaffold technology. Finally, this study aims to provide a proof-of-concept screening of the intrinsic and synergistic antimicrobial potential of these biopolymers against a broad spectrum of resistant pathogens, comparing the efficacy of the dual-product scaffold against pure HA and complex HA-Moringa oil emulsions to establish a foundation for next-generation bioactive wound dressings.

## 2. Materials and Methods

### 2.1. Concurrent Fermentation of HA and Polyhydroxybutyrate (PHB)

Following the successful optimization of hyaluronic acid (HA) production by *H. bluephagenesis* TD01 harboring the pMCSeSD-*araBAD*-*pmHasA* gene, which yielded a molecular weight (Mw) of 9.6 × 10^6^ Da in the modified 40LBG-Y medium (yeast extract as a sole nitrogen source). A one-step fermentation process was developed to evaluate the simultaneous production of HA and polyhydroxybutyrate (PHB) by this strain in response to the L-arabinose inducer. This study aimed to characterize the metabolic relationship between HA biosynthesis, PHB accumulation, and total cellular biomass. Fermentation was conducted using modified 40-LBG-Y medium, as previously identified as the optimal medium for HA production in this chassis [8]. The culture conditions were maintained at pH 8.0, 25 °C, and 250 rpm using the L-arabinose induction system for a total duration of 72 h.

### 2.2. Biomass, Sample Preparation, and HA Extraction

After 72 h of cultivation, 50 mL of fermentation broth was harvested and partitioned for subsequent analysis: 10 mL was dedicated to HA extraction and quantification [8], while the remaining 40 mL was utilized for PHB and biomass assays. The cell pellets were collected via centrifugation at 10,000× *g* for 15 min, washed twice with 20 mL of distilled water, and frozen at −80 °C for a minimum of 1 h. Samples were then lyophilized for over 24 h. Cell growth was quantified as Cell Dry Weight (CDW, g/L). To differentiate between total biomass and the intracellular bioplastic, True Cell Mass (TCM, g/L) was calculated by subtracting the PHB titer from the total CDW [12].

### 2.3. Analysis of PHB Content and Titer

Intracellular PHB content was quantified using Gas Chromatography (GC-2014, Shimadzu, Kyoto, Japan). Approximately 40–50 mg of lyophilized cell mass underwent acid-catalyzed methanolysis to convert the PHB polymer into its constituent methyl ester monomers. The reaction mixture consisted of 2 mL of chloroform and 2 mL of esterifying solution (3% H_2_SO_4_ and 1 g/L benzoic acid in methanol). The mixture was heated at 100 °C for 4 h. After cooling to room temperature (25 °C), 1 mL of distilled water was added to the samples. Following vigorous vortexing and phase separation, 1 mL of the lower chloroform phase was sampled for GC analysis. Commercial Poly(R)-3-hydroxybutyric acid (99.9%, Sigma-Aldrich, St. Louis, MO, USA) served as the external standard. PHB metric was defined as follows: PHB Titer (g/L): PHB content (wt%) × CDW.

### 2.4. Extraction and Film Formation

For physical characterization, intracellular PHB was extracted using a Soxhlet extractor (Soxtec 2050, Foss, Hillerød, Denmark). The extracted polymer was dissolved in chloroform and subsequently precipitated in a 10-fold volume of absolute ethanol. Following centrifugation at 12,000 rpm for 10 min, the precipitates were re-dissolved in chloroform and cast onto plates. Solvent evaporation resulted in the formation of thin PHA/PHB films for further analysis.

### 2.5. Preparation of HA-PHB Nanoparticle Scaffold Composites

#### 2.5.1. Synthesis of PHB Nanoparticles (NPs)

**Figure 2:**
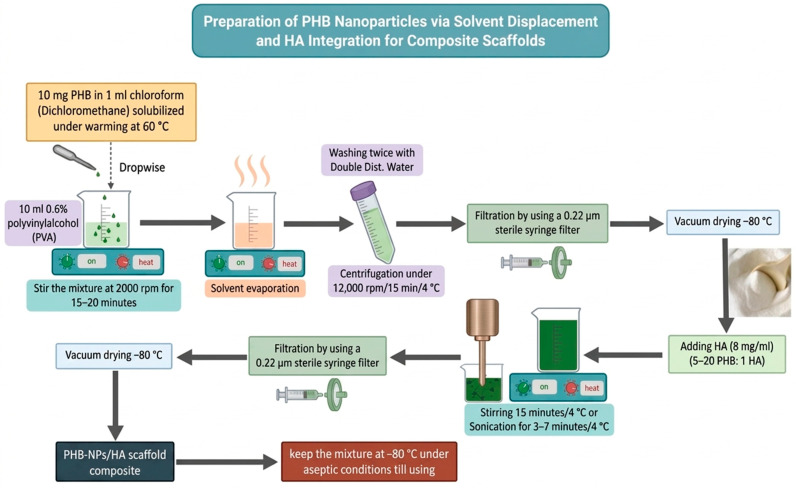
Schematic workflow for the preparation of PHB nanoparticles and their subsequent integration with hyaluronic acid (HA) to form a composite scaffold.

Polyhydroxyalkanoate (PHB) nanoparticles were formulated via the solvent displacement method, as previously described [55,56,57,58]. Briefly, 10 mg of PHA was dissolved in 1 mL of dichloromethane (DCM) under intense stirring to ensure complete homogenization. This organic phase was then added dropwise into 10 mL of 0.6% polyvinyl alcohol (PVA) serving as a stabilizer under magnetic stirring at 800 rpm. Following a 10-min stabilization period, the emulsion was maintained under stirring in a fume hood overnight to allow for the complete evaporation of the dichloromethane. The resulting nanoparticles were collected by centrifugation at 12,000 rpm for 15 min at room temperature, washed twice with ultra-pure water to remove residual surfactant, and finally re-dispersed in ultra-pure water (Figure 2).

#### 2.5.2. Formulation of the HA-PHB Composite Scaffold

To prepare the composite scaffold, hyaluronic acid (HA) was integrated into the PHA matrix [55,56,57,58]. Hyaluronic acid was added to the PHB-NPs organic solution and stirred until completely dissolved. The weight ratio of PHB to hyaluronic acid was maintained within a range of 5–20:1. The mixture was processed to obtain microspheres through evaporation and subsequent drying. The final composite was freeze-dried for a minimum of 23 h to produce a stable, porous powder. The resulting HA-PHB nanoparticle scaffold composites were stored in a freezer until further experimental use (Figure 2).

### 2.6. Characterization of the HA-PHB/NP Scaffold Composites

#### 2.6.1. Morphological Analysis

The surface morphology and structural integrity of the HA-PHB blend films and scaffolds were characterized using Scanning Electron Microscopy (SEM) (Zeiss EVO 18; ZEISS SUPRA-55 SAPPHIRE, Oberkochen, Germany). Before imaging, samples were processed to ensure conductivity, allowing for high-resolution visualization of the composite’s surface topography and the distribution of nanoparticles within the hyaluronic acid matrix.

#### 2.6.2. Particle Size and Zeta Potential

The hydrodynamic diameter, particle size distribution, and polydispersity index (PDI) of the PHA-NPs and HA-PHA/NP composites were determined via Dynamic Light Scattering (DLS) using a Malvern Zetasizer Nano-ZS (Malvern, Worcestershire, UK). Furthermore, the surface charge and colloidal stability of the nanoparticles were evaluated by measuring the Zeta potential [59]. All measurements were conducted in triplicate at room temperature to ensure statistical reliability and to assess the influence of hyaluronic acid integration on the overall stability of the PHA nanoparticles.

### 2.7. Preparation of HA-Moringa Oil Seed Extract (MO/HA)

The preparation of the Moringa oil and hyaluronic acid (MO/HA) complex followed a two-step process: first, formulating an oil-in-water (O/W) emulsion, then integrating it with HA. Moringa oil (MO) was procured from Greentec Imaging Co., Ltd., Jiaxing, China. Initially, MO was blended with Tween-80 at a 1:1 (*v*/*v*) ratio. In this system, Tween-80 functions as a nonionic surfactant to stabilize the o/w emulsion and facilitates the efficient solubilization of the hydrophobic oil into the aqueous phase. This MO/Tween-80 mixture was then added to an HA solution (8 mg/mL) and subjected to continuous magnetic stirring for 6 h to ensure the formation of a stable, homogeneous complex. Finally, the resulting MO/HA solution was purified by filtration through a 0.22 µm polyethersulfone membrane to ensure sterility and remove any undispersed particulates [60].

### 2.8. Antimicrobial Evaluation of HA-PHB/NP Blends and MO/HA Extracts

#### 2.8.1. Microbial Strains and Culture Conditions

The antimicrobial activity was evaluated against six bacterial strains and one fungal strain: *Klebsiella variicola* ATCC 488, *Pseudomonas aeruginosa* ATCC 15442, *Staphylococcus aureus* ATCC 25923, *Aeromonas hydrophila* ATCC 35654, *Shigella sonni* ATCC 29930, *Cronobacter sakazakii* ATCC 29544, and the yeast *Candida albicans* ATCC 90028, which were obtained from the Institute of Mitochondrial Biology and Medicine, Xi’an Jiaotong University (IMBM, Xi’an, China). Microbial stocks preserved at −80 °C were revitalized in Brain Heart Infusion (BHI) broth for bacteria and Sabouraud Dextrose Broth (SDB) for fungi. Purity was verified by streaking onto selective agar plates. Isolated colonies were used to inoculate subcultures, which were incubated for 18 h (bacteria) or 24 h (yeasts) to reach mid-exponential phase.

#### 2.8.2. Standardization of Inocula

Initial microbial concentrations were adjusted to approximately 3.4 × 10^8^ CFU/mL for bacteria and 2.1 × 10^6^ CFU/mL for fungi, corresponding to the optical density (O.D.) of a 0.5 McFarland standard [61]. Working suspensions were then prepared in their respective media to achieve the following target concentrations: *S. sonni*: 3.5 × 10^8^ CFU/mL, *C. sakazakii*: 2.6 × 10^8^ CFU/mL, *A. hydrophila*: 3.1 × 10^8^ CFU/mL, *K. variicola*: 3.3 × 10^7^ CFU/mL, *P. aeruginosa*: 3.26 × 10^7^ CFU/mL, *S. aureus*: 2.49 × 10^7^ CFU/mL, and *C. albicans*: 2.1 × 10^5^ CFU/mL. To ensure precision, the volume of culture required to prepare 1 mL of standardized inoculum was calculated using the following equation: *V_culture_* (µL) = 1000 µL/*OD*_measured_/*OD*_target_, where *OD* measured was recorded at 600 nm for bacteria and 540 nm for yeast. Internal validation was performed via viable cell counting (CFU/mL) to confirm the accuracy of the densitometric measurements. Densitometric assays were conducted using (Thermo MULTISKAN Spectrum, Thermo Fisher Scientific, Waltham, MA, USA), with McFarland standards serving as the reference for all standardized inocula [62].

#### 2.8.3. Microplate Antimicrobial Assay

The antimicrobial assays were conducted in 96-well microplates (Corning Inc., Corning, NY, USA) to evaluate the inhibitory effects of the HA-based blends and extracts. All test preparations were sterilized via filtration through a 0.22 µm membrane before inoculation. Microbial suspensions (150 µL/well) were combined with 150 µL of the respective treatment or control solutions to achieve the following final concentrations (Table 1).

The plates included blank wells containing only BHI broth and saline to account for background absorbance. Additionally, 200 µL of McFarland standards were dispensed into specific wells to serve as internal densitometric controls. Every experimental condition was tested in triplicate. The microplates were incubated at 37 °C in a moist chamber to prevent evaporation. Optical density (*OD*) measurements were recorded at time 0 (immediately following inoculation) and after 24 h of incubation. Bacterial growth was monitored at 600 nm, while yeast growth was measured at 540 nm using a Thermo Multiskan Spectrum microplate reader (Thermo Fisher Scientific, Waltham, MA, USA). To correlate absorbance values with actual microbial viability, a Colony Forming Units (CFUs) assay was performed at the 24 h mark. Samples were randomly collected from the wells, serially diluted, and plated onto agar to determine the viable count.

### 2.9. Statistical Analysis

Experimental data were obtained from three independent biological replicates (*n* = 3 independent biological replicates) and are presented as the mean ± standard deviation (SD). The homogeneity of variances was confirmed, and statistical differences between groups were analyzed using one-way Analysis of Variance (ANOVA), followed by Tukey’s post-hoc test for multiple comparisons. Comparisons between groups were performed using an unpaired Student’s *t*-test. A *p*-value of less than 0.05 was considered statistically significant (* *p* < 0.05, ** *p* < 0.01, *** *p* < 0.001), and ns = not significant (*p* > 0.05).

## 3. Results

### 3.1. One-Step Concurrent Production of HA and PHB

**Figure 3:**
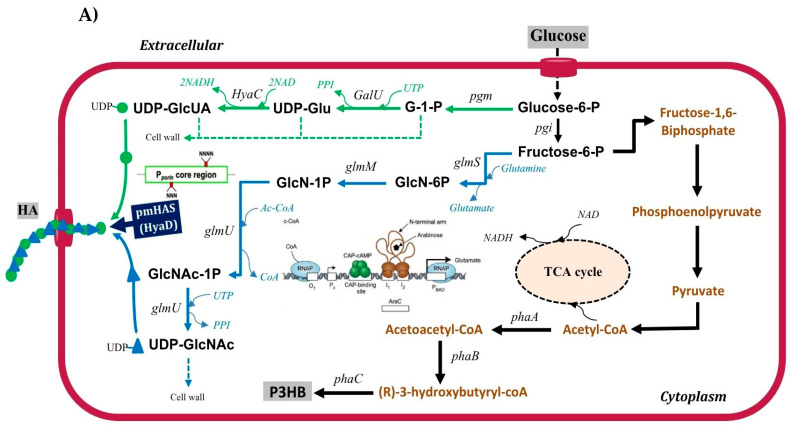

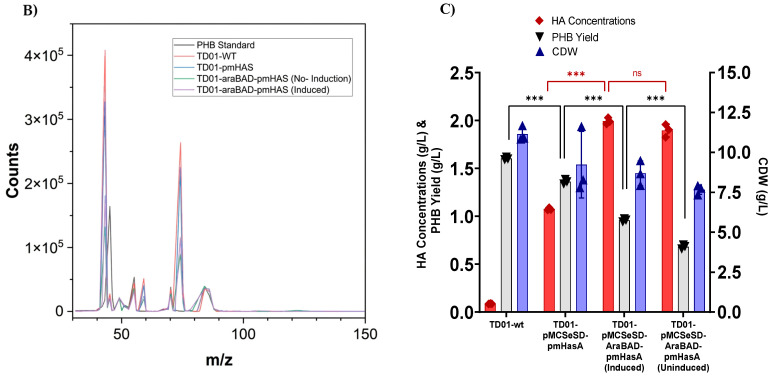
Recombinant *H. bluephagenesis* TD01 for HA and PHB co-production. (**A**) The metabolic network illustrates the redirection of glucose flux into three primary routes: the synthesis of UDP-glucuronic acid (UDP-GlcUA, green arrows), the hexosamine biosynthetic pathway for UDP-N-acetylglucosamine (UDP-GlcNAc, blue arrows), and toward acetyl-CoA for P3HB production (black arrows) via the phaABC operon. A heterogeneous fine-tuned biosynthesis pathway for enhancing the HA production and metabolic fluxes by gene *pmHasA*, cloned from *Pasteurella multocida*, which encodes HA synthase, under the *araBAD* promoter, is shown in green and blue bold lines. HA chains are elongated by HA synthase (pmHAS; HyaD), from two building blocks: UDPGlcA (uridine diphosphate glucuronate) and UDP-GlcNAc (uridine diphosphate N-acetylglucosamine). Pgm, phosphoglucomutase; GalU, glucose-1-phosphate uridylyltransferase; hyaC, UDP-glucose 6-dehydrogenase; Pgi, glucose-6-phosphate isomerase; GlmS, L-glutamine-D-fructose-6-phosphate aminotransferase; GlmM, phosphoglucosamine mutase; GlmU, UDP-N-acetylglucosamine pyrophosphorylase/Glucosamine-1-phosphate N-acetyltransferase. (**B**) Mass spectra (*m*/*z* vs. counts) of cell extracts from the wild-type strain (TD01-WT), the *pmHasA*-expressing strain, and the inducible TD01-pMCSeSD-*araBAD*-*pmHasA* strain (induced and uninduced), compared against a commercial P3HB standard. The overlapping peaks at characteristic *m*/*z* values confirm the chemical identity of the intracellularly accumulated polymer across all engineered variants, along with HA (confirmation of HA production as reported in [60]. (**C**) Determination of HA concentration, P3HB yield, and cell dry weight (CDW) among the wild-type (TD01-wt) and engineered strains (TD01-pMCSeSD-*pmHasA* and TD01-pMCSeSD-*araBAD*-*pmHasA* under induced and uninduced conditions) in modified 40-LBG-Y rich medium. Statistical significance by two-way ANOVA is indicated with the *p* value between groups, which is indicated by asterisks (***, *p* < 0.0001), while ‘ns’ denotes a non-significant difference. The error bars represent standard deviations of triplicates (*n* = 3).

Building upon previous findings that identified *H. bluephagenesis* TD01-pMCSeSD-*araBAD*-*pmHasA* as the most efficient enzyme for hyaluronic acid (HA) biosynthesis and best optimized conditions for HA production [8], the current study utilized this strain to evaluate a simplified, cost-effective fermentation process. The engineered *H. bluephagenesis* TD01 was cultivated in nutrient-rich 40-LBG-Y medium for HA production, and partitioning carbon flux from glucose-6-phosphate into three distinct pathways (Figure 3A). The rich 40-LBG-Y environment supported significantly higher biomass and HA titers across all recombinant variants and facilitated the simultaneous biosynthesis of hyaluronic acid (HA) with poly(3-hydroxybutyrate) (P3HB). The convergence of these two nucleotide sugars in the presence of the hyaluronan synthase (*pmHasA*) enabled the polymerization and subsequent extracellular secretion of HA (green and blue pathway). Concurrently, downstream glycolytic flux was directed toward the formation of acetyl-CoA, which was diverted from the tricarboxylic acid (TCA) cycle to the P3HB biosynthetic pathway (orange-brown pathway). This route utilizes the phaABC enzymatic sequence to convert acetyl-CoA into (R)-3-hydroxybutyryl-CoA, leading to the intracellular accumulation of P3HB. A regulatory module sensitive to arabinose and cAMP levels was integrated to balance the metabolic demands between precursor availability and polymer synthesis.

To verify the production of P3HB within the engineered strains, mass spectrometric analysis was performed on polymer extracts and compared to a P3HB standard (Figure 3B). The mass spectra revealed identical fragmentation patterns between the standard and the biological samples, with prominent peaks observed at characteristic mass-to-charge ratios (*m*/*z*), notably around 43 and 86, which correspond to the expected monomeric fragments of the polyester. While P3HB production was confirmed in all tested samples, the peak intensities varied significantly between strains. The TD01-wt strain exhibited the highest counts, indicating robust native P3HB accumulation. In contrast, the strains engineered for HA synthesis (TD01-pMCSeSD-*pmHasA* and the inducible *araBAD* variant) showed a measurable decrease in peak intensity. This reduction suggests a redistribution of the shared acetyl-CoA pool toward the hexosamine and UDP-GlcUA biosynthetic pathways for HA production. Furthermore, the TD01-pMCSeSD-*araBAD*-*pmHasA* strain demonstrated a distinct shift in P3HB levels upon induction.

Quantitative analysis of the fermentation products revealed a distinct metabolic tradeoff between HA synthesis and P3HB accumulation (Figure 3C, Table 2). All engineered strains showed a highly significant increase in HA production compared to the wild type (*p* < 0.001). The comparison between the TD01-wild-type (0.0869 g/L) and the engineered strains represents a highly significant increase in HA production (*p* < 0.001). The induced TD01-pMCSeSD-*araBAD*-*pmHasA* strain produced more HA (1.99 g/L) than the uninduced (1.89 g/L), as a noticeable trend toward higher yield upon induction. The induced TD01-pMCSeSD-*araBAD*-*pmHasA* strain with L-arabinose vs. the uninduced strain comparison was not significant (“ns”) (*p* = 0.085, >0.05). The SEM values are extremely low (all below 0.04), indicating that the mean estimates are very precise and that the experimental technique is highly reproducible. Also, the introduction of the HA production pathways significantly reduced the PHB content across all engineered strains compared to the wild-type TD01-wt (1.60 ± 0.014, *p* < 0.001), confirming that the redirection of carbon flux toward the nucleotide sugar precursors occurs at the expense of the acetyl-CoA pool required for P3HB. Notably, induction with L-arabinose in the TD01-pMCSeSD-*araBAD*-*pmHasA* strain resulted in a significantly higher PHB (0.958 ± 0.016) compared to the uninduced TD01-pMCSeSD-*araBAD*-*pmHasA* strain (0.681 ± 0.020, *p* < 0.001), highlighting the efficacy of the P*_BAD_* regulatory system in modulating the metabolic tradeoff between the two biopolymers. Furthermore, the engineered strains showed a significant reduction in CDW relative to the wild-type (TD01-wt), likely due to the metabolic burden associated with co-producing HA and PHB. The metabolic burden of producing HA and PHB appears to cause a measurable decrease in biomass compared to the wild-type (WT). While the induced group shows a higher mean CDW (8.69 g/L) than the uninduced group (7.67 g/L), the difference is not quite statistically significant (*p* = 0.099). *H. bluephagenesis* TD01 has been extensively engineered to produce small molecules in one step, such as ectoine (28 g/L in fed-batch), 5-aminovaleric acid (67.4 g/L), and 3-hydroxypropionate (154 g/L), and other products [63]. The metabolic versatility of *H. bluephagenesis* TD01 as a chassis for co-production was evaluated by comparing the performance of the HA/PHB system with other high-value bioproducts reported in the literature.

### 3.2. Physical and Therapeutic Characterization of the HA-PHB/NP Scaffold Composite

**Figure 4:**
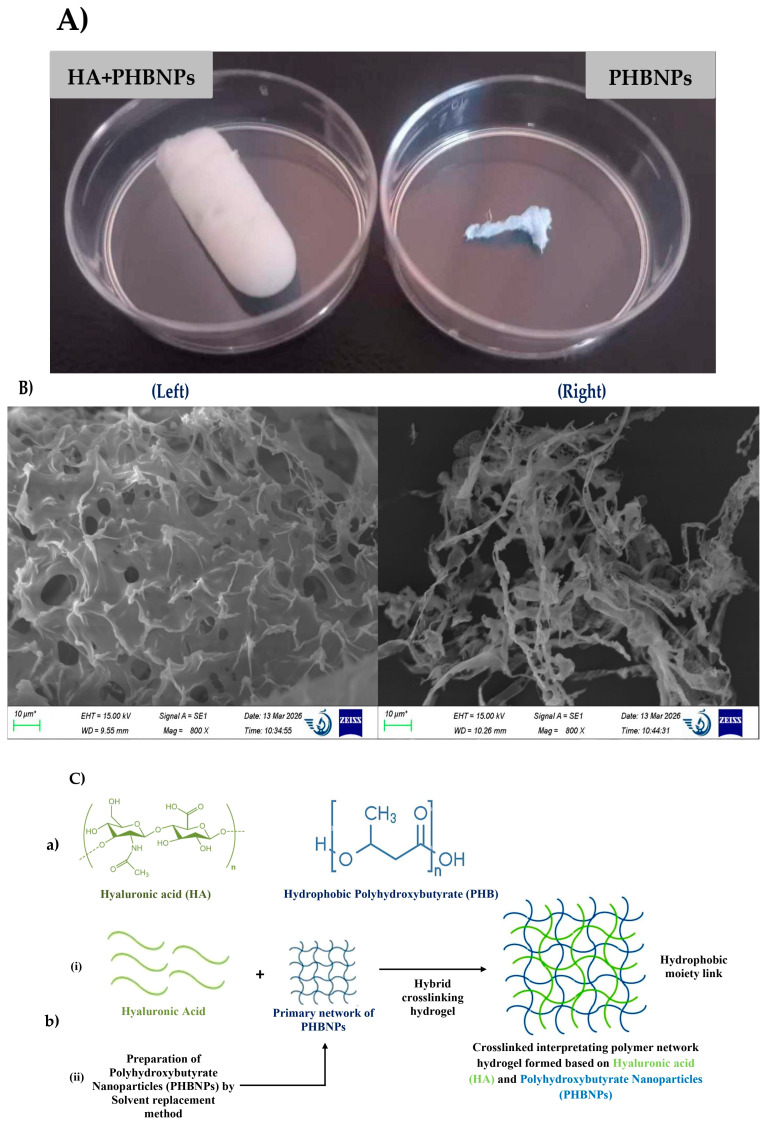
Morphological and mechanistic analysis of HA-PHB nanoparticle scaffolds. (**A**) Macro-scale morphology: a digital photograph of the HA + PHB nanoparticle (NP) and pure PHB-NP scaffolds illustrates the initial physical differences between the blended composite and the pure nanoparticle base. (**B**) Microstructural SEM Analysis: Scanning electron microscopy (SEM) provides a comparative visualization of scaffold morphologies at 800× magnification (accelerating voltage: 15.00 kV; * scale bar: 10 µm). The pure PHB-NP scaffold (right panel) exhibits a disjointed and predominantly fibrous morphology. In contrast, the HA-PHBNPs scaffold (left panel) displays a well-defined, highly porous, and interconnected three-dimensional network. The integration of hyaluronic acid with PHB nanoparticles ensures the fabrication of a cohesive biomaterial scaffold suitable for further functional studies. (**C**) A schematic model illustrates the proposed dissolution, dispersion, and assembly of polyhydroxybutyrate nanoparticles (PHB-NPs) within a hyaluronic acid (HA) matrix. (**a**) Chemical Structures of the Precursors: Displays the respective chemical structures of the repeating polymeric units for both hydrophilic hyaluronic acid (HA) and hydrophobic polyhydroxybutyrate (PHB). (**b**) Schematic Illustration of Hybrid Network Formation: Outlines the two-step fabrication process governed by specific hydrophilic and hydrophobic interactions during the mixing phase: (i) Linear HA chains are integrated with a (ii) primary network of PHB-NPs, which are synthesized via the solvent replacement method. This blending process yields a crosslinked, interpenetrating polymer network (IPN) stabilized by hydrophobic-moiety linkages. The underlying mechanism of this hydrogel formation and its dual-component dispersion is interpreted based on [64,65,66].

The fabricated HA-PHB scaffold composite exhibited a white, porous macroscopic appearance, physically resembling a plastic sponge-like fiber. Notably, the composite prepared at a 1:20 (HA: PHB) ratio was semi-transparent, as shown in Figure 4A.

### 3.3. Characterization of HA-PHB/NP Composites via SEM and Zeta Potential

To investigate the structural integration and morphological properties of the synthesized biopolymers, the surface architecture of the HA-PHB/NP blend was examined using Scanning Electron Microscopy (SEM).

#### 3.3.1. Comparative Morphological Analysis

The architectural differences between the pure PHB nanoparticle-based scaffold and the HA-PHB composite are significant. The PHB nanoparticles alone (Figure 4A, Right) tended to form a fragmented, fibrous, and loosely organized structure. In contrast, the incorporation of hyaluronic acid (Figure 4B, Left) facilitated the development of a highly porous, interconnected three-dimensional network. This sponge-like scaffold morphology suggests that the HA functions as a cohesive matrix, effectively bridging the PHB nanoparticles to create a stable environment with a high specific surface area (Figure 4C). As illustrated, this porous architecture is considered an optimal configuration for biological scaffolds.

#### 3.3.2. Functional Implications for Antimicrobial Potential Use

The observed interconnected porosity is particularly advantageous for the intended antimicrobial applications. Such a structure may significantly enhance the potential loading capacity for therapeutic agents and allow for the more precise, controlled release of bioactive compounds (Figure 4). Furthermore, the high surface-area-to-volume ratio provided by the HA-matrixed nanoparticles suggests improved interaction with the surrounding biological environment, which is critical for effective wound management and infection control.

#### 3.3.3. Particle Size Distribution and Architectural Transformation

**Figure 5:**
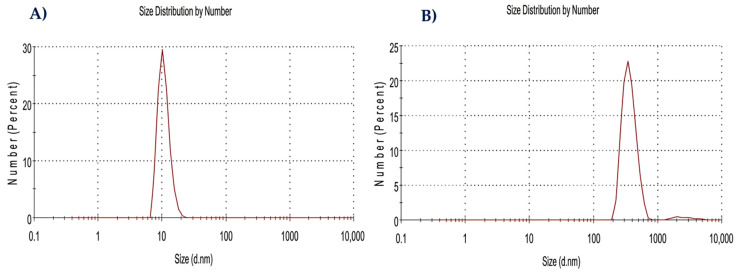
Size distribution analysis of PHB and HA-PHB nanoparticles measured by a Zeta potential analyzer. The plots show the number-based size distribution of the synthesized nanoparticles, with Panel (**A**) showing pure PHB nanoparticles (PHBNPs) with a peak diameter of approximately 12 nm, indicating a high degree of monodispersity. Panel (**B**) illustrates the shift in distribution for the HA-PHB nanoparticle composite (HA-PHBNPS), which exhibits an average diameter of 450 nm. This marked increase in particle size serves as physical evidence for the successful integration of hyaluronic acid into the PHB nanoparticle system, which is essential for the subsequent formation of the three-dimensional HA-PHB scaffolds.

To characterize the physical dimensions of the primary building blocks used in scaffold fabrication, the hydrodynamic diameter and size distribution of the pure PHB nanoparticles (PHBNPs) and the hyaluronic acid-blended composite (HA-PHBNPs) were analyzed via Dynamic Light Scattering (DLS). As illustrated in Figure 5, the pure PHBNPs exhibited a highly uniform and narrow size distribution, with a mean hydrodynamic diameter of 12 nm (Figure 5A). This small, consistent size profile is characteristic of efficiently stabilized PHB cores. However, upon the incorporation of HA, a dramatic shift in the size profile was observed; the HA-PHBNPs displayed a significantly larger average diameter of 450 nm (Figure 5B). This nearly 40-fold increase in diameter confirms the successful coating and complexation of the high-molecular-weight HA polymer with the PHB nanoparticle cores. This substantial size transition indicates that HA is not merely dispersed within the medium but is fundamentally integrated into the particle architecture, likely through the formation of a thick hydration shell or a polymer-matrix network. This transition from individual 12 nm particles to larger 450 nm composite aggregates provides the essential structural foundation for the interconnected porous network observed in the final 3D scaffold.

### 3.4. Antimicrobial Properties of the HA-PHB/NP Scaffold and MO/HA Extract

#### 3.4.1. Inhibitory Effects of the HA-PHB/NP Scaffold Composite

##### Antifungal Activity Against *Candida albicans*

In *C. albicans*, the antimicrobial action of the engineered nanomaterial formulations was evaluated against a control population (Figure 5A and Table 3). As shown in the statistical profile, pure HA (0.914 ± 0.024) and pure PHBNPs (0.944 ± 0.026) exhibited no significant inhibitory variations when compared against the control group (0.873 ± 0.066, *p* > 0.05). Conversely, the composite formulation HA-PHBNPs demonstrated a prominent and highly significant antimicrobial action (0.679 ± 0.033), driving a significant value reduction compared to the control (*p* < 0.01), pure HA, and pure PHBNPs (*p* < 0.001). The composite HA-PHBNPs exhibited strong antimicrobial activity compared to all other groups (*p* < 0.001). Tukey’s HSD post-hoc analysis confirmed that HA-PHBNPs significantly reduced the target value compared to the untreated control. Conversely, no significant differences were observed for the remaining pairwise comparisons (HA vs. control and PHBNPs vs. control, *p* > 0.05), with both groups performing similarly to the negative control.

##### Antibacterial Activity Against *Klebsiella variicola*

**Figure 6:**
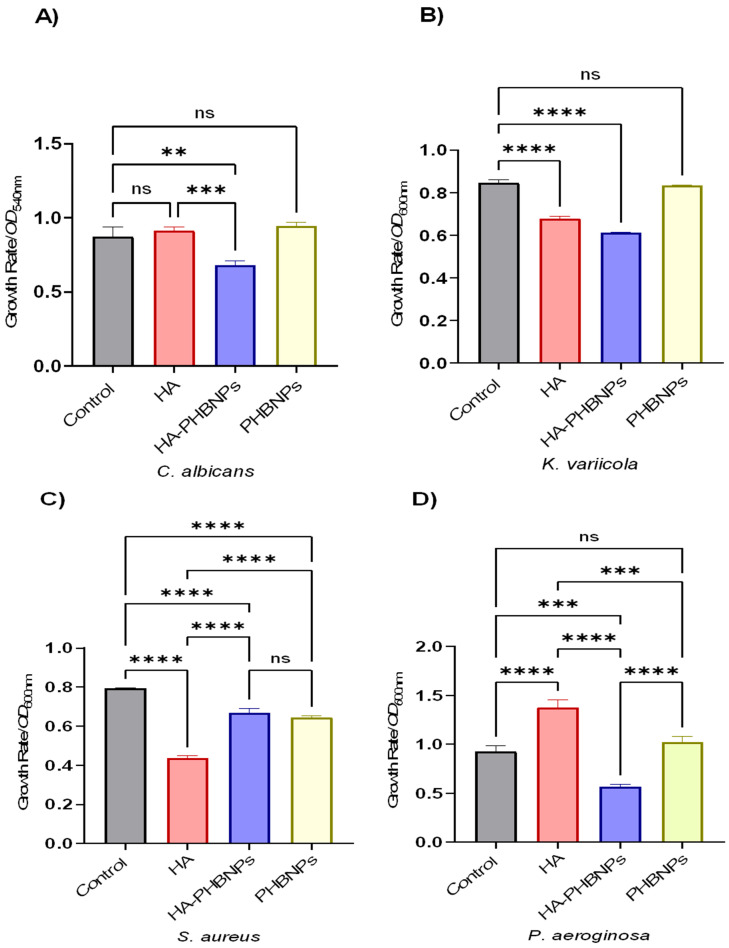
Preliminary screening efficacy of the engineered HA-PHBNP scaffold against: (**A**) *C. albicans*, the bar graph illustrates the growth rate was monitored at *OD*_540nm_, (**B**) *K. variicola*, (**C**) *S. aureus*, (**D**) *P. aeruginosa*, the bar graph represents the growth rate under (*OD*_600nm_). All treatments were under four conditions: Control, purified HA, HA-PHBNP composite, and purified PHBNPs only, and their concentrations are mentioned in Table 1. Statistical significance is indicated by asterisks (**** for *p* < 0.0001), (*** for *p* < 0.001), (** for *p* < 0.01), and “ns” denotes no significant difference relative to the control. Data are expressed as the mean of three independent replicates, with error bars representing the standard deviation.

The antimicrobial efficiency of the nanomaterial formulations against *K. variicola* revealed pronounced variations among the treatments (Figure 6B and Table 3). While pure PHBNPs (0.832 ± 0.004) demonstrated no distinct inhibitory capacity compared to the baseline Control (0.846 ± 0.016), the introduction of pure HA yielded a highly significant reduction (0.678 ± 0.012). Crucially, the composite HA-PHBNPs formulated group achieved the highest level of antimicrobial action (0.612 ± 0.002), proving significantly superior to both the untreated control and its separate individual components, pure HA and pure PHBNPs.

The composite HA-PHBNPs exerted the strongest overall inhibitory impact against *K. variicola*, significantly reducing the target value compared to the untreated control (*p* < 0.001), pure HA (*p* = 0.0002), and pure PHBNPs (*p* < 0.001). Pure HA also demonstrated distinct antimicrobial potential relative to the control (*p* < 0.001). Conversely, pure PHBNPs yielded no significant change against the control (*p* = 0.417), behaving identically to the untreated baseline. The superior efficacy of the composite formulation over both individual components indicates a functional synergy, likely attributed to increased surface area and enhanced structural stability.

##### *Staphylococcus* *aureus*

The testing of nanomaterial variants against the gram-positive bacterium *S. aureus* displayed strong, highly significant inhibitory profiles across all treated parameters (Figure 6C and Table 3). In comparison to the base control population (0.794 ± 0.003), structural reductions were achieved by pure PHBNPs (0.645 ± 0.008) and the composite matrix HA-PHBNPs (0.668 ± 0.024), with no statistically significant performance variations distinguishing the two nanoparticle variations from one another (*p* > 0.05). Strikingly, pure HA demonstrated the highest degree of antimicrobial potential action against *S. aureus*, reducing the metric value down to 0.436 ± 0.013. This performance was highly significant compared to the baseline control, pure PHBNPs, and the blended HA-PHBNPs formulation.

The variance is highly significant (*p* < 0.0001), confirming that differences among the treatment setups are not due to random chance. Every single tested substance (HA, HA-PHBNPs, and PHBNPs) caused a highly significant drop in values relative to the untreated control (*p* < 0.001). Unlike the trends observed in the Gram-negative bacterial data, pure HA demonstrated the strongest inhibitory action against *S. aureus*, significantly outperforming both the pure PHBNPs (*p* < 0.001) and the composite HA-PHBNPs matrix (*p* < 0.001). HA-PHBNPs vs. PHBNPs showed no statistically significant difference between the composite nanoparticles and the pure nanoparticles (*p* = 0.2709, ns). They suppress *S. aureus* growth to a similar degree. *S. aureus* (Gram-positive) shows a unique effect to pure Hyaluronic Acid (HA), whereas embedding HA into the PHB nanoparticle matrix actually slightly lessened its single-agent activity against this specific strain.

##### *Pseudomonas* *aeruginosa*

In the testing of nanomaterial variants against *P. aeruginosa*, the engineered biomaterial platforms exhibited highly distinct profiling changes (Figure 6D and Table 3). While treatment with pure PHBNPs (1.019 ± 0.061) showed no statistically significant divergence from the untreated control (0.924 ± 0.060), exposure to pure HA led to a substantial value elevation (1.374 ± 0.080, *p* < 0.001). Most importantly, the composite matrix HA-PHBNPs produced the most intense antimicrobial performance (0.565 ± 0.027), yielding an extremely significant reduction when evaluated against the baseline control, pure PHBNPs, and pure HA.

The overall variations among sample populations are highly statistically significant (*p* < 0.0001). For HA-PHBNPs vs. control, the value significantly dropped by 0.3592 (*p* = 0.0004). This statistically establishes the composite formulation’s intense inhibitory capabilities against *P. aeruginosa*. Pure HA actually showed a significant increase in the measured value (*p* = 0.0001). Pure HA may be promoting or serving as a matrix, making the massive drop found in the composite even more remarkable.

Pure PHBNPs do not demonstrate a statistically significant change (*p* = 0.2896), acting roughly equivalent to the baseline control. The composite formulation HA-PHBNPs significantly outperforms pure HA (*p* < 0.0001) and pure PHBNPs (*p* = 0.0001), solidifying a stark formulation synergy. All the HA, HA-PHBNPs, and PHBNPs concentrations used are mentioned in Table 1.

#### 3.4.2. Antimicrobial Evaluation of the HA-Moringa Oil (MO/HA) Complex

##### *Aeromonas* *hydrophila*

**Figure 7:**
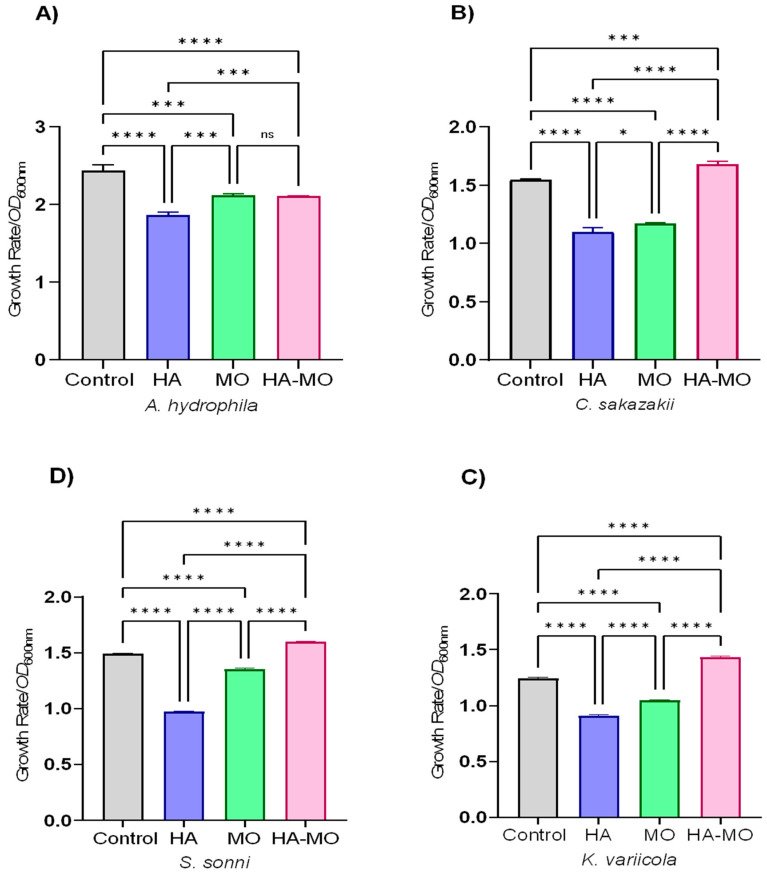
Preliminary screening activity of HA, MO, and HA-MO complexes against: (**A**) *A. hydrophila*, (**B**) *C. sakazakii*, (**C**) *K. variicola*, and (**D**) *S. sonni*. The inhibitory potential of purified HA, Moringa oil (MO), and the HA-MO complex was evaluated by monitoring the growth rate through (*OD*_600nm_). All concentrations used are mentioned in Table 1. Statistical significance is indicated by asterisks (**** for *p* < 0.0001), (*** for *p* < 0.001), (* for *p* < 0.05), and “ns” denotes no significant difference relative to the control. Data are expressed as the mean of three independent replicates, with error bars representing the standard deviation.

Screening of biomaterial variants against *A. hydrophila* revealed highly significant, distinctive growth inhibition across all treatment protocols compared to the uninhibited control population (2.430 ± 0.082) (Figure 7A and Table 4). Exposure to pure MO (2.119 ± 0.019) and the composite matrix HA-MO (2.103 ± 0.010) both yielded highly significant, equivalent levels of growth containment (*p* < 0.001 vs. control), with no statistical deviation found between the two particle formulations. Notably, pure HA demonstrated the most prominent anti-microbial efficacy against this strain, reducing the growth parameter metric to (1.867 ± 0.030). This baseline reduction was found to be highly statistically significant when evaluated against the untreated Control, pure MO, and the hybrid HA-MO blend.

The overall variations across the groups are highly statistically significant (*p* < 0.0001), showing that the differences between the tested treatments are clear and reliable. The HA, MO, and the HA-MO composite resulted in a highly significant drop in growth compared to the untreated control group (*p* < 0.0001) compared by using Tukey’s HSD test. Pure HA demonstrated the most robust antimicrobial profile against *A. hydrophila*. It significantly outperformed the untreated control (*p* < 0.001), pure MO (*p* = 0.0006), and the combined HA-MO matrix (*p* < 0.0009). There is no statistically significant difference between the composite HA-MO formulation and pure MO (*p* = 0.9694, ns). Blending them yielded practically the same inhibition profile as the pure MO component on its own.

##### *Cronobacter* *sakazakii*

Extensive screening against the pathogen *C. sakazakii* highlighted distinct bi-directional shifts in growth responses across the varied treatment matrices relative to the baseline control (1.545 ± 0.008) (Figure 7B and Table 4). Both pure HA (1.097 ± 0.037) and pure MO (1.173 ± 0.003) exhibited robust antimicrobial behavior, yielding highly significant suppressions in microbial metrics, with HA proving marginally more suppressive than MO (*p* = 0.0108). Conversely, the formulation of the hybrid HA-MO composite matrix completely reversed this trend, triggering a highly significant increase in growth values up to 1.683 ± 0.020 (*p* < 0.001 against control, HA, and MO populations). This points to an interactive masking phenomenon within the composite layout that effectively neutralizes the baseline potency of its individual constituents. The overall variance between the group means is exceptionally significant (*p* < 0.0001), confirming that the structural shifts in *C. sakazakii* density across groups are highly reproducible and not due to chance. This indicates a fascinating antagonistic or structural masking effect. While both raw hyaluronic acid and the MO component strongly inhibit *C. sakazakii* when applied individually, combining them into the HA-MO composite eliminates this antimicrobial property. Instead, the composite network appears to act as a supportive substrate or nutrient source that slightly promotes *C. sakazakii* proliferation relative to the control.

##### *Klebsiella* *variicola*

Screening protocols against *K. variicola* revealed highly significant, bi-directional growth alterations across the evaluated treatment matrices when compared to the untreated control group (1.244 ± 0.007) (Figure 7C and Table 4). Both pure HA (0.910 ± 0.011) and pure MO (1.047 ± 0.004) demonstrated highly effective antimicrobial action, generating highly significant suppressions in microbial density parameters, with pure HA providing the most pronounced baseline inhibition. In contrast, the application of the integrated HA-MO composite matrix completely reversed this behavior, causing a highly significant increase in final growth parameters up to 1.433 ± 0.010 (*p* < 0.001 relative to Control, HA, and MO populations). This structural turnaround suggests a complex masking interaction within the co-polymer framework that deactivates the baseline antimicrobial properties of its individual constituents.

##### *Shigella* *sonni*

Screening protocols against the pathogen *S. sonnei* revealed highly significant (Figure 7D and Table 4), bi-directional growth alterations across all evaluated treatment matrices when compared to the untreated control group (1.495 ± 0.001). Both pure HA (0.972 ± 0.003) and pure MO (1.354 ± 0.008) demonstrated highly effective antimicrobial capabilities, generating highly significant suppressions in microbial density parameters (*p* < 0.001), with pure HA providing the most pronounced baseline inhibition. In contrast, the application of the integrated HA-MO composite matrix completely reversed this behavior, causing a highly significant increase in final growth parameters up to 1.597 ± 0.009 (*p* < 0.001 relative to control, HA, and MO populations). This structural turnaround highlights a profound masking interaction within the co-polymer framework that deactivates the baseline antimicrobial properties of its individual constituents. All treatments were under four conditions: control, purified HA, MO, and HA-MO composite, and their concentrations are mentioned in Table 1.

## 4. Discussion

In this study, we aimed to elucidate the metabolic relationship between hyaluronic acid (HA) and polyhydroxybutyrate (PHB) biosynthesis in *Halomonas bluephagenesis* TD01 (Figure 3A). This system relies on the strategic manipulation of intracellular carbon flux in the optimized 40-LBG-Y medium, which was previously identified as superior for high-titer HA production [67,68]. While the engineered strains showed a significant increase in HA production over the wild type, arabinose induction only marginally shifted yields from 1.89 g/L to 1.99 g/L. Conversely, introducing the HA pathway drastically decreased PHB content across all engineered strains compared to the TD01-wt baseline of 1.60. This drop confirms that shifting carbon flux toward nucleotide sugars (the HA precursors UDP-GlcUA and UDP-GlcNAc) depletes the acetyl-CoA pool required for PHB synthesis, highlighting the metabolic burden inherent in dual-polymer co-production [69]. Similarly, Yang et al. [70] and Choi et al. [71] used engineered E. coli strains for the production of aromatic (52.3 mol% 3-hydroxybutyrate (3HB)-co-47.7 mol% d-phenyllactate), and poly(lactate-co-glycolate) from glucose by one-step fermentation.

To balance these competing metabolic demands, a regulatory module (porine, a cellular membrane protein that acts as a pore, through which molecules can diffuse) sensitive to arabinose and cAMP levels was integrated. Interestingly, L-arabinose induction in the TD01-pMCSeSD-*araBAD*-*pmHasA* strain yielded significantly higher PHB (0.958) than its uninduced counterpart (0.681), demonstrating that the *P_BAD_* regulatory system effectively modulates the metabolic tradeoff between the two polymers (Figure 3C and Table 2). However, the baseline, non-induced expression of this system also provides a highly productive and balanced metabolic flux that supports simultaneous cell growth and polymer synthesis. The finding that this uninduced state performs nearly as well as the induced state in 40-LBG-Y medium represents a major advantage for Next-Generation Industrial Biotechnology (NGIB). By leveraging high baseline expression in robust media, industrial production processes can be simplified and operational costs significantly reduced by eliminating the need for expensive chemical inducers, as was mentioned by Sukan et al. [72].

Structural confirmation of the PHB monomers extracted from the bacterial biomass further demonstrated the successful co-production of PHA alongside HA. Similarly, Chen et al. [11] confirmed PHB production by *H. bluephagensis* TD01. This confirms that *H. bluephagenesis* TD01 can function as a dual-product factory, potentially enhancing the economic viability of the fermentation process (Figure 3C). Also, this comparative data suggests that the HA/PHB co-production platform is a competitive addition to the growing portfolio of *Halomonas*-based biomanufacturing strategies [6,63,73,74,75,76] (Table 5).

After extraction and characterization of HA and PHB, the purified, high-molecular-weight HA was blended with PHB nanoparticles (PHB/NPs) to fabricate a composite nanoparticle scaffold. SEM imaging confirmed the integrated morphology of the HA-PHB/NP structure. Similarly, our results were matched with Bhattacharya et al. [76], who reported that SEM data showed an increase in particle size (<200 nm) after hyaluronic acid conjugation over polymeric nanoparticles.

This sponge-like architecture suggests that HA acts as a cohesive matrix, effectively bridging individual PHB nanoparticles to create a stable, high-surface-area environment, an observation that aligns with findings reported by Ladhari et al. [77] and Ahmed et al. [78] (Figure 4). This physical crosslinking strategy is highly beneficial for biomedical applications, as it eliminates the need for potentially toxic chemical crosslinking agents and allows it to respond to environmental stimuli [79]. The formation of the HA-based hydrogel matrix is driven primarily by hydrophobic interaction pathways, a combination of non-covalent interactions, including ionic bonds, hydrogen bonding, and van der Waals forces, which facilitates robust hydrophobic self-assembly [80]. Also, for an interconnected 3D hydrogel network (Figure 4C), a porous framework is highly advantageous for the intended antimicrobial applications, as it significantly enhances the loading capacity and the controlled release kinetics of bioactive compounds [64,65,81,82].

Similarly, as noted by Tenje et al. [82], the matrix determines the matrix’s ability to facilitate the adequate exchange of active therapeutic agents and cellular debris within the microenvironment and for pharmaceutical applications. Also, the hydrophobic domains within the HA-PHBNP matrix provide stable sequestered environments for drug loading, while the hydrophilic HA network ensures the scaffold maintains its biocompatibility and high swellability [83] (Figure 4A, left). Similarly, this was demonstrated in previous reports using HA-interpenetrating networks for luteolin delivery [84]. The transition was from 12 nm (pure PHB/NPs) to 450 nm (composite HA-PHB/NPs). These results, which are matched with Bhattacharya et al. [76] (Figure 5), are highly significant, and a substantial shift in hydrodynamic diameter further supports this physical integration. It clearly demonstrates that the high-molecular-weight HA is not merely dispersed near the nanoparticles but fundamentally alters the particle architecture through extensive complexation.

The biological potential of the *Halomonas*-derived HA was rigorously validated through preliminary antimicrobial assays against representative Gram-positive, Gram-negative, and yeast strains. The experimental data demonstrate that the HA-PHB/NP scaffold exhibits potent antimicrobial activity, showing significant efficacy against fungal pathogens (*C. albicans*), Gram-negative bacteria (*K. variicola* and *P. aeruginosa*), and Gram-positive bacteria (*S. aureus*). This bioactivity was quantified by significant reductions in both OD_600nm_ and corresponding colony-forming units (CFU/mL) (Figure 6). Several key mechanisms documented in the literature support the efficacy of the HA-PHBNP scaffold. First, the intrinsic protective properties of HA produced by *H. bluephagenesis* TD01 provide a functional barrier against clinical infections, aligning with reports that HA inhibits Pseudomonas species [85] and a wide range of standard ATCC microbial strains [61]. While PHB is traditionally recognized as a structural skeleton for HA, recent studies suggest that raw PHB particles can independently exhibit antimicrobial activity against common skin pathogens, including *Staphylococcus* epidermidis and *C. albicans* [86]. Interestingly, our results contrast with these findings, as the standalone PHB particles did not show significant antimicrobial effects. Ultimately, the synergy between the bioactive HA matrix and the structural contributions of the PHB nanoparticles yields a prospective, multi-targeted antimicrobial effect. This consistent performance across diverse microbial models underscores the potential of the HA-PHBNP system as an advanced scaffold for wound healing and protective biomedical coatings.

The most compelling finding in our antimicrobial assays was observed with *C. albicans* (Figure 6A), where purified HA failed to inhibit growth. This lack of inhibition is likely because approximately 97.8% of *C. albicans* clinical isolates are prolific producers of hyaluronidase [87]. In contrast, *K. variicola* and *S. aureus*, which often utilize HA-containing capsules as virulence factors rather than metabolic substrates, proved more susceptible to the intrinsic antimicrobial properties of the purified HA (Figure 6B,C) [88]. A similar trend was observed in the divergent response of *P. aeruginosa* to the purified HA versus the HA-PHBNP scaffold (Figure 6D). This phenomenon may be elucidated by the high enzymatic potency of *P. aeruginosa*, which produces approximately 130 U/mg of hyaluronidase, a significantly higher concentration than that observed in many other bacterial species [89,90]. This elevated hyaluronidase activity allows *P. aeruginosa* to rapidly degrade the standalone HA polymer, utilizing it as a carbon substrate or leveraging the hydrogel as a protective niche for proliferation. However, when HA is integrated into the PHB nanoparticle matrix, its structural configuration is fundamentally altered. We hypothesize that the dense, physically crosslinked network of the scaffold sterically hinders the hyaluronidase from accessing the polymer’s glycosidic bonds [91]. This configuration effectively masks the polymer from enzymatic degradation, transforming a potential nutrient source into a resilient antimicrobial barrier, though this proposed mechanism warrants further experimental validation.

Furthermore, preliminary screening confirmed that pristine PHB nanoparticles (PHB-NPs) exhibit no inherent antimicrobial activity against the tested pathogens. This finding aligns with recent literature indicating that while PHB serves as an excellent biocompatible carrier for therapeutic agents, the polymer itself lacks independent bactericidal properties [92,93]. Thus, the composite’s high efficacy is likely driven by the structural stabilization of bioactive HA by the PHB matrix rather than any intrinsic activity from the PHB itself. This structural integration minimizes enzymatic degradation and maximizes HA interactions with the bacterial cell wall.

We propose that several distinct physicochemical mechanisms contribute to the scaffold’s consistent, versatile performance across diverse microbial environments: The negatively charged HA matrix induces strong steric and electrostatic repulsion against the similarly negatively charged bacterial membranes. This creates an “antifouling” effect that prevents initial bacterial adhesion and subsequent colonization, contrasting sharply with the behavior of cationic hydrophilic polymers like chitosan [94]. By acting as a specialized artificial barrier, the dense HA-PHB-NP composite physically restricts microbial proximity to the protected surface, preventing bio-interfacial contact. The high molecular weight (Mw) of HA within the nanoparticle scaffold induces hyaluronidase saturation. By providing an excess of substrate, the system overwhelms bacterial enzymatic capacity, rendering the microbes unable to efficiently depolymerize the matrix [22,95]. While these coordinated structural and electrostatic defenses explain the scaffold’s resilience, further experimental validation is underway to fully map these interfacial interactions.

Additionally, when evaluated against a panel of pathogens including *A. hydrophila*, *C. sakazakii*, *K. variicola*, and *S. sonnei*, the *Halomonas*-derived HA demonstrated superior antimicrobial efficacy compared to both the pure Moringa oil (MO) and the MO/HA composite (Figure 7A–D). While certain strains like *A. hydrophila* and *K. variicola* are known hyaluronidase producers, they were still significantly inhibited by the high-molecular-weight HA (Mw-HA). This efficacy is likely because the high concentration of bio-engineered HA overwhelms bacterial hyaluronidases, preventing complete hydrolysis and maintaining the polymer’s inhibitory framework, a stark contrast to the low HA concentrations typically found in human tissue [96,97].

A provocative finding was that the MO/HA composite resulted in higher *OD*_600nm_ readings than the control group for *C. sakazakii*, *K. variicola*, and *S. sonnei*, suggesting an antagonistic relationship or metabolic interference within the emulsion. We propose three primary mechanisms to account for this observed growth promotion: The pathogens may metabolically utilize the Moringa oil or the non-ionic surfactants within the emulsion as primary carbon sources, fueling accelerated growth; the emulsion matrix may act as a physical barrier that traps the active components, severely limiting their immediate release and bioavailability at the bio-interface; and the surfactant and oil droplets may physically disrupt the conformation of the HA polymer, hindering its ability to saturate bacterial hyaluronidases or exert the necessary steric and electrostatic repulsions against the bacterial cell wall.

The consistent failure of the MO/HA composite to outperform pure HA across these diverse strains underscores the fact that this high-quality, bio-engineered HA is a superior and more reliable antimicrobial candidate than the oil-based formulation. Consequently, future development of these biomaterials should focus on optimizing the delivery of pure *Halomonas*-derived HA to preserve its maximum therapeutic potential.

Ultimately, leveraging the robust *Halomonas*-chassis within a ‘Next-Generation Industrial Biotechnology’ (NGIB) framework enables highly efficient heterologous expression of the *pmHasA* gene. This process yields a high-molecular-weight polymer with exceptional bioactivity and structural quality, making it a premier candidate for topical administration. These results emphasize that the synthesized HA is not merely a generic biopolymer but a high-performance functional material [25,98]. Its intrinsic ability to outperform established natural extracts, such as Moringa oil, highlights its value for antimicrobial coatings. Furthermore, given its high biocompatibility and potent inhibitory profile against ESKAPE pathogens (including *P. aeruginosa* and *S. aureus*), this premium HA serves as an ideal foundation for the HA-PHB/NP antibiotic delivery system against multidrug-resistant strains [99]. Further studies evaluating MIC, MBC, and time-kill data are warranted to confirm these mechanisms and assess the system’s full potential as a treatment for multidrug-resistant pathogens.

## 5. Conclusions

The successful development of a one-step fermentation process for the co-production of hyaluronic acid (HA) and polyhydroxybutyrate (PHB) marks a significant milestone in metabolic engineering and industrial sustainability. By utilizing the robust *H. bluephagenesis* TD01 platform, this research effectively bypasses the safety risks and high purification costs associated with traditional pathogenic hosts, establishing a non-toxic and economically viable production model. The study highlighted that strategic manipulation of intracellular carbon flux is needed for the efficient partitioning of glucose into dual high-value polymers without imposing a prohibitive metabolic burden on the microbial chassis, so we suggest more studies to maximize the productivity for both polymers.

The engineered strains represent a highly significant increase in HA production. The induced TD01-pMCSeSD-*araBAD*-*pmHasA* strain group produced more HA (1.99 g/L) than the uninduced strain (1.89 g/L), with a noticeable trend toward higher yield upon induction. Introducing HA production pathways significantly reduced PHB content across all engineered strains compared to the wild-type TD01-wt (1.60 g/L), confirming that diverting carbon flux toward nucleotide sugars depletes the acetyl-CoA pool needed for PHB. Notably, L-arabinose induction of the TD01-pMCSeSD-*araBAD*-*pmHasA* strain yielded significantly higher PHB (0.95 g/L) than the uninduced control (0.681 g/L). This demonstrates the efficacy of the PBAD regulatory system in balancing the metabolic tradeoff between these two biopolymers.

The sponge-like architecture suggests that HA acts as a cohesive matrix, effectively bridging individual PHB nanoparticles to create a stable, high-surface-area environment. Fabricating this material into a physically crosslinked, 3D porous network provides a critical structural masking effect. This steric shielding restricts bacterial hyaluronidases from accessing and rapidly degrading the HA, preventing *P. aeruginosa* and similar pathogens from exploiting the polymer as a nutrient source. Consequently, the scaffold transforms a typically vulnerable nutrient into a robust antimicrobial barrier. The biological potential of this sophisticated biomaterial was confirmed through preliminary assays, where the *Halomonas*-derived HA-PHB scaffold demonstrated potent antimicrobial efficacy against *C. albicans*, *K. variicola*, *P. aeruginosa*, and *S. aureus*, showing significant reductions in both OD600 and colony-forming units (CFU/mL). Furthermore, the *Halomonas*-derived HA independently demonstrated greater antimicrobial activity against *A. hydrophila*, *C. sakazakii*, *K. variicola*, and *S. sonnei* than both pure Moringa oil (MO) and the MO/HA composite.

Furthermore, the rigorous comparative analysis highlights the superior antimicrobial potential of pure, *Halomonas*-derived HA compared with complex natural extracts such as Moringa oil. Our findings demonstrate that this *Halomonas*-produced HA, synthesized as a biopolymer via the NGIB approach, consistently performs the ‘heavy lifting’ in antimicrobial assays. Ultimately, we hypothesize that the HA-PHB system provides an ideal, sustainable platform for next-generation wound dressings and protective biomedical coatings, offering a powerful strategy to combat the global challenge of antimicrobial resistance (AMR). Further studies evaluating MIC, MBC, and time-kill kinetics are warranted to confirm these mechanisms and assess the system’s full potential against multidrug-resistant pathogens.

## Figures and Tables

**Table 1:** HA, PHB-NPs, and Moringa oil concentrations and preparation in this study.

Group	Composition	Final Concentration
Control	Saline Solution	0.9% NaCl
HA	Hyaluronic Acid	8 mg/mL
HA + PHB-NPs	HA-PHBNPs Blend	8 mg/mL (HA) + 10 mg/mL (NPs)
PHB-NPs	PHB Nanoparticles	10 mg/mL
HA-MO	HA + Moringa Oil	(8 mg/mL HA 1:1 *v*/*v* MO)
MO	Moringa Oil	1:1 *v*/*v* (in Tween-80)

**Table 2:** Quantitative analysis of hyaluronic acid (HA), cell dry weight (CDW), and poly(3-hydroxybutyrate) (PHB) production across engineered *H. bluephagenesis* TD01 strains in 40-LBG-Y medium.

Media Type		Production in 40-LBG-Y ^1^				
Strains			TD01-WT	TD01-pMCSeSD-*pmHasA*	TD01-pMCSeSD-*araBAD*-*pmHasA* (Induced)	TD01-pMCSeSD-*araBAD*-*pmHasA* (Uninduced) ^2^
Product	HA	HA conc. Mean (g/L)	0.086	1.075	1.993	1.895
		HA conc. (g/L) ± SD	0.08 ± 0.002	1.07 ± 0.012	1.99 ± 0.032	1.89 ± 0.066
		SEM (±)	±0.001	±0.007	±0.019	±0.038
		HA Sig. (vs. WT)	-	***	***	***
	CDW	CDW Mean (g/L)	11.14	9.23	8.69	7.67
		CDW (g/L) ± SD	11.13 ± 0.47	9.23 ± 2.08	8.69 ± 0.77	7.66 ± 0.30
		SEM (±) of CDW	±0.271	±1.203	±0.447	±0.173
		CDW Sig. (vs. WT)	-	ns	**	***
	PHB	PHB yield Mean (g/L)	1.602	1.360	0.958	0.681
		PHB Yield (g/L) ± SD	1.60 ± 0.01	1.36 ± 0.02	0.95 ± 0.01	0.68 ± 0.02
		SEM (±)	0.008	0.013	0.009	0.011
		PHB Sig. (vs. WT)	-	***	***	***

^1^ 40-LBG-Y: (Modified Luria Bertani; 40 g/L NaCl; 20 g/L Glucose; 25.22 g/L Yeast extract). Data are expressed as mean ± SD and SEM (*n* = 3) for HA concentrations, CDW, and PHB yield, models. Significance levels denote variations against the control group (*H. bluephagenesis* TD01-WT) using a one-way ANOVA with Tukey’s post-hoc test (ns: *p* > 0.05; ** *p* < 0.001; *** *p* < 0.0001). ^2^ uninduced strains: no addition of L-arabinose in the used medium, and culture conditions were pH: 8.0, 250 rpm, 25 °C.

**Table 3:** Antimicrobial activity evaluation and statistical significance of HA, PHBNPs, and HA-PHBNPs formulations.

Strain	Substance/Treatment ^1^	Bacterial Growth Density (*OD*) Mean ^2^	SD (±)	SEM (±)	Significance (vs. Control)
*C. albicans*	Control	0.8729	0.0664	0.0383	—
	HA	0.9136	0.0241	0.0139	ns
	HA-PHBNPs	0.6788	0.0327	0.0189	****
	PHBNPs	0.9436	0.0256	0.0148	ns
*K. variicola*	Control	0.8457	0.0158	0.0091	—
	HA	0.6778	0.0120	0.0069	***
	HA-PHBNPs	0.6124	0.0016	0.0009	***
	PHBNPs	0.8323	0.0036	0.0021	ns
*S. aureus*	Control	0.7940	0.0029	0.0017	—
	HA	0.4357	0.0125	0.0072	***
	HA-PHBNPs	0.6679	0.0237	0.0137	***
	PHBNPs	0.6452	0.0079	0.0046	***
*P. aeruginosa*	Control	0.9244	0.0602	0.0348	—
	HA	1.3744	0.0799	0.0461	*** (Increase)
	HA-PHBNPs	0.5652	0.0274	0.0158	*** (Inhibition)
	PHBNPs	1.0190	0.0607	0.0350	ns

^1^ Data are expressed as mean ± SD and SEM (*n* = 3) for Gram-negative, Gram-positive, and fungal microbial models. Significance levels denote variations against the untreated Control group using a one-way ANOVA with Tukey’s post-hoc test (ns: *p* > 0.05; *** *p* < 0.001; **** *p* < 0.0001). ^2^ *OD*_540nm_ for *C. albicans* and *OD*_600nm_ for bacteria. All the HA, HA-PHBNPs, and PHBNPs concentrations are mentioned in Table 1.

**Table 4:** Bacterial growth densities (*OD*_600nm_), standard deviations, standard errors, and significance levels of four distinct bacterial strains under control, monomeric (HA, MO), and composite (HA-MO) treatment environments.

Strains	Substance/Treatment ^1^	Bacterial Growth Density Mean ^2^	SD (±)	SEM (±)	Significance (vs. Control)
*A. hydrophila*	Control	2.4303	0.0822	0.0475	—
	HA	1.8667	0.0297	0.0172	***
	MO	2.1193	0.0190	0.0110	***
	HA-MO	2.1031	0.0095	0.0055	***
*C. sakazakii*	Control	1.5454	0.0079	0.0046	—
	HA	1.0970	0.0371	0.0214	*** (Inhibition)
	MO	1.1728	0.0033	0.0019	*** (Inhibition)
	HA-MO	1.6831	0.0197	0.0114	*** (Growth promotion)
*K. variicola*	Control	1.2440	0.0071	0.0041	—
	HA	0.9103	0.0105	0.0061	*** (Inhibition)
	MO	1.0475	0.0041	0.0024	*** (Inhibition)
	HA-MO	1.4328	0.0105	0.0061	*** (Growth promotion)
*S. sonni*	Control	1.4948	0.0012	0.0007	—
	HA	0.9723	0.0028	0.0016	*** (Inhibition)
	MO	1.3536	0.0080	0.0046	*** (Inhibition)
	HA-MO	1.5973	0.0085	0.0049	*** (Growth promotion)

^1^ The baseline values, variations (±SD), and precision metrics (±SEM) are calculated from three technical replicates. Statistical thresholds are marked relative to the control population (*** *p* < 0.001; highly significant), with directional modulation noted explicitly in parentheses where distinct phenotypic reversals (inhibition vs. growth promotion) occurred. ^2^ Cell Density Index measured at *OD*_600nm_. All treatments were under four conditions: control, purified HA, purified MO only, and HA-MO composite; their concentrations are mentioned in Table 1.

**Table 5:** Comparative analysis of co-production platforms using engineered *Halomonas bluephagenesis* TD01 under different culture conditions.

Strain	Culture Mode	Culture Medium	Aeration Parameters	Co-Production Yields		References
				PHB	Co-Product	
*H. bluephagenesis* TD01 harboring (*pmHasA* with *P_araBAD_* promoter)	Batch 250 mL	40-LBG-Y (20 g/L glucose, 25.22 g/L yeast extract, 40 g/L NaCl, pH 8–9)	200–250 rpm	PHB: 0.68–1.60 g. L^−1^	HA:1–1.99 g. L^−1^ Mw: 9.67 × 10^6^ Dalton	This study
*H. bluephagenesis* TD01 harbors genes encoding L-lysine monooxygenase (DavB) and 5-aminovaleramide amidohydrolase (DavA).	Batch and fed-batch 7 L bioreactor	3 L 50 MM supplemented with 2 g. L^−1^ K_2_HPO_4_, 5 g. L^−1^ yeast extract, 20 g. L^−1^ glucose and 1 g. L^−1^ urea	DO%30%1 VVM <800 rpm, 37 °C	PHA: 42 wt%	5-Aminovaleric Acid (5-AVA)/5-Hydroxyvalerate (5HV): batch culture 16.4 g L^−1^ and Fed-batch; 67.4 g. L^−1^ 5-AVA (4.8 mol%)	[73]
*H. bluephagenesis* TD01 harboring hmgCAB cassette encoding fatty acid photodecarboxylase (CvFAP) and hydroxymandelic acid synthase (SyHMAS).	Fed-batch	For propane LB-60 pH 6.8. For Mandelate and hydroxymandelate: LB-60 pH 6.7, tyrosine (0–3 g/L), phenylalanine (0–3 g/L), glucose (0–30 g/L), phenylpyruvate (10–20 mM), and/or glycerol (0–10 g/L). For PHA, LB60 pH 9 containing 15 g. L^−1^ glucose, or modified high salt MM-63 medium pH 9 containing 10 g/L glucose.	For Propane: 180 rpm under a 455 nm LED blue light panel, 30 °C for 10 h. For Mandelate and hydroxymandelate: 30 °C at 180 rpm for 48 h. For PHA: 30 °C with 180 rpm agitation	up to 72 g/g DCW %	Biofuel: Propane and mandelate 62 g/g DCW and 71 ± 10 mg/L, respectively	[74]
*H. bluephagenesis* TD01 harboring aldehyde dehydrogenase (AldDHb), 3HP degradation pathway deletion, and overexpression of alcohol dehydrogenases (AdhP).	Fed-batch, 7 L bioreactor	Solution I containing 800 g. L^−1^ glucose and 30 g. L^−1^ urea. Solution II containing 800 g. L^−1^ glucose and 15 g. L^−1^ and 60 g. L^−1^ 1,3-propanediol and 6 g. L^−1^ acetic acid were added three times separately	37 °C, 200 to 800 rpm	PHB: 60%wt%	3-Hydroxypropionate (3HP): ~12 g/L 3HP: 154 g. L^−1^/45%. 0.93 g. g^−1^ 1,3-propanediol and 2.4 g. L^−1^ h^−1^ (32–45%)	[75]
*H. bluephagenesis* TD01 using codon-optimized gene encoding α-amylase from *Bacillus licheniformis*	Batch 250 mL	Corn starch 60-MMG and 60-MMS media supplemented with 100 mM sodium aspartate, 100 mM KCl, and 1.7 g. L^−1^ citric acid. Adding 5 g. L^−1^ of γ-butyrolactone, starch, and glucose.	48 h, pH 8–9	PHA: 9.5 g/L CDW consisting of 52 wt% PHB	Extracellular Enzymes (Amylase): N.R.	[63]
*H. bluephagenesis* harboring ectABC, lysC, and asd, and bypasses deletion.	Fed-batch 7 L bioreactor	60-MM medium supplemented with 3 g. L^−1^ CO(NH_2_)_2_, 10 g. L^−1^ yeast extract, and 20 g. L^−1^ glucose, non-sterilized, open condition at 37 °C. pH 9.0	44 h growth, (DO%) was maintained at ~30%, 1 VVM 800 rpm	PHB: 32 g. L^−1^ dry cell mass containing 75% PHB	Ectoine: 8 g. L^−1^ (Batch); 28 g. L^−1^ (Fed-batch)	[6]

## Data Availability

The original contributions presented in this study are included in the article/Supplementary Materials. Further inquiries can be directed to the corresponding author.

## Supplementary Materials

The following supporting information can be downloaded at: https://www.mdpi.com/article/10.3390/biom16060889/s1, Figure S1: Mass spectra (*m*/*z* vs. counts) of cell extracts for PHB characterization by the *H. bluephagenesis* TD01 wild-type strain (TD01-WT), TD01-pmHAS strain (2LB), and TD01-*araBAD-pmHAS* strains (induced 3LB and non-induced 4LB) [8], compared against a commercial P3HB standard (Std).

## References

[1] Serra M., Casas A., Toubarro D., Barros A.N., Teixeira J.A. (2023). Microbial Hyaluronic Acid Production: A Review. Molecules.

[2] Bhagowati P., Pradhan S., Dash H.R., Das S. (2015). Production, Optimization, and Characterization of Polyhydroxybutyrate, A Biodegradable Plastic, by *Bacillus* spp. Biosci. Biotechnol. Biochem..

[3] Shalin T., Sindhu R., Binod P., Soccol C.R., Pandey A. (2013). Mixed Cultures Fermentation for The Production of Poly-ß-Hydroxybutyrate. Braz. Arch. Biol. Technol..

[4] Pan N.C., Pereira H.C., da Silva M.D., Vasconcelos A.F., Celligoi M.A. (2017). Improvement Production of Hyaluronic Acid by *Streptococcus zooepidemicus* in Sugarcane Molasses. Appl. Biochem. Biotechnol..

[5] Zhang Y., Dong J., Xu G., Han R., Zhou J., Ni Y. (2023). Efficient Production of Hyaluronic Acid by *Streptococcus zooepidemicus* Using Two-Stage Semi-Continuous Fermentation. Bioresour. Technol..

[6] Ma H., Zhao Y., Huang W., Zhang L., Wu F., Ye J., Chen G.Q. (2020). Rational Flux-Tuning of *Halomonas bluephagenesis* for Co-Production of Bioplastic PHB and Ectoine. Nat. Commun..

[7] Tan B., Zheng Y., Yan H., Liu Y., Li Z.J. (2022). Metabolic Engineering of *Halomonas bluephagenesis* to Metabolize Xylose for Poly-3-Hydroxybutyrate Production. Biochem. Eng. J..

[8] Marwan-Abdelbaset E., Lu X., Tan D. (2026). Engineering *Halomonas bluephagenesis* TD01 as a robust chassis for the sustainable production of Hyaluronic acid. Biomolecules.

[9] Marwan-Abdelbaset E., Samy-Kamal M., Tan D., Lu X. (2025). Microbial Production of Hyaluronic Acid: The Current Advances, Engineering Strategies and Trends. J. Biotechnol..

[10] Sun S., Yang S., Qiu Y., Ding J., Wang W., Wu F., Chen G.Q. (2025). Life Cycle Design of Polyhydroxyalkanoates (PHA). Natl. Sci. Rev..

[11] Chen X., Yin J., Ye J., Zhang H., Che X., Ma Y., Li M., Wu L.P., Chen G.Q. (2017). Engineering *Halomonas bluephagenesis* TD01 For Non-Sterile Production of Poly (3-Hydroxybutyrate-Co-4-Hydroxybutyrate). Bioresour. Technol..

[12] Tan D., Xue Y.S., Aibaidula G., Chen G.Q. (2011). Unsterile and Continuous Production of Polyhydroxybutyrate by *Halomonas* TD01. Bioresour. Technol..

[13] Wang X., Liu X., Li C., Li J., Qiu M., Wang Y., Han W. (2025). Effects of Molecular Weights on The Bioactivity of Hyaluronic Acid: A Review. Carbohydr. Res..

[14] Caon I., Parnigoni A., Viola M., Karousou E., Passi A., Vigetti D. (2021). Cell Energy Metabolism and Hyaluronan Synthesis. J. Histochem. Cytochem..

[15] DeAngelis P.L., Zimmer J. (2023). Hyaluronan Synthases; Mechanisms, Myths, & Mysteries of Three Types of Unique Bifunctional Glycosyltransferases. Glycobiology.

[16] Xie J.H., Xin J.Y., Sun L.R., Cui T.Y., Bi H.X., Wang Y., Zhang J.X. (2025). The Role of Biological Nitrogen Fixation in Polyhydroxybutyrate Production from Methane by Methane-Oxidizing Bacteria: A Review of Metabolic Routes and Yield Enhancement. Front. Microbiol..

[17] McAdam B., Brennan Fournet M., McDonald P., Mojicevic M. (2020). Production of Polyhydroxybutyrate (PHB) And Factors Impacting Its Chemical and Mechanical Characteristics. Polymers.

[18] Agüero-Chapin G., Antunes A., Marrero-Ponce Y. (2026). A 2026 Update on Computational Approaches to the Discovery and Design of Antimicrobial Peptides. Antibiotics.

[19] Iaconisi G.N., Lunetti P., Gallo N., Cappello A.R., Fiermonte G., Dolce V., Capobianco L. (2023). Hyaluronic Acid: A Powerful Biomolecule with Wide-Ranging Applications-A Comprehensive Review. Int. J. Mol. Sci..

[20] Tavianatou A.G., Caon I., Franchi M., Piperigkou Z., Galesso D., Karamanos N.K. (2019). Hyaluronan: Molecular Size-Dependent Signaling and Biological Functions in Inflammation and Cancer. FEBS J..

[21] Nicolosi B., Kruszewska K., Gonçalves V. (2025). Hyaluronic Acid in Wound Management: An Update on Formulations, Mechanisms, and Clinical Applications. Int. J. Wound Res..

[22] Zamboni F., Wong C.K., Collins M.N. (2022). Hyaluronic Acid Association with Bacterial, Fungal, and Viral Infections: Can Hyaluronic Acid Be Used as An Antimicrobial Polymer for Biomedical and Pharmaceutical Applications?. Bioact. Mater..

[23] Poletajew S., Brzózka M.M., Krajewski W., Kamecki H., Nyk Ł., Kryst P. (2024). Glycosaminoglycan Replacement Therapy with Intravesical Instillations of Combined Hyaluronic Acid and Chondroitin Sulfate in Patients with Recurrent Cystitis, Post-radiation Cystitis and Bladder Pain Syndrome: A Narrative Review. Pain Ther..

[24] Touati A., Mairi A., Ibrahim N.A., Idres T. (2025). Essential Oils for Biofilm Control: Mechanisms, Synergies, and Translational Challenges in the Era of Antimicrobial Resistance. Antibiotics.

[25] Alipoor R., Ayan M., Hamblin M.R., Ranjbar R., Rashki S. (2022). Hyaluronic Acid-Based Nanomaterials as A New Approach to The Treatment and Prevention of Bacterial Infections. Front. Bioeng. Biotechnol..

[26] Bayer I.S. (2020). Hyaluronic Acid and Controlled Release: A Review. Molecules.

[27] Yoon M.S., Lee J.M., Jo M.J., Kang S.J., Yoo M.K., Park S.Y., Bong S., Park C.S., Park C.W., Kim J.S. (2025). Dual-Drug Delivery Systems Using Hydrogel–Nanoparticle Composites: Recent Advances and Key Applications. Gels.

[28] Kaniuk Ł., Stachewicz U. (2021). Development and Advantages of Biodegradable PHA Polymers Based on Electrospun PHBV Fibers for Tissue Engineering and Other Biomedical Applications. ACS Biomater. Sci. Eng..

[29] Marcello E., Nigmatullin R., Basnett P., Maqbool M., Prieto M.A., Knowles J.C., Boccaccini A.R., Roy I. (2024). 3D Melt-Extrusion Printing of Medium Chain Length Polyhydroxyalkanoates and Their Application as Antibiotic-Free Antibacterial Scaffolds for Bone Regeneration. ACS Biomater. Sci. Eng..

[30] Lezcano M.F., Álvarez G., Chuhuaicura P., Godoy K., Alarcón J., Acevedo F., Gareis I., Dias F.J. (2022). Polyhydroxybutyrate (PHB) Scaffolds for Peripheral Nerve Regeneration: A Systematic Review of Animal Models. Biology.

[31] Shlapakova L.E., Botvin V.V., Mukhortova Y.R., Zharkova I.I., Alipkina S.I., Zeltzer A., Dudun A.A., Makhina T., Bonartseva G.A., Voinova V.V. (2024). Magnetoactive Composite Conduits Based on Poly(3-hydroxybutyrate) and Magnetite Nanoparticles for Repair of Peripheral Nerve Injury. ACS Appl. Bio Mater..

[32] Perveen K., Masood F., Hameed A. (2020). Preparation, Characterization, And Evaluation of Antibacterial Properties of Epirubicin-Loaded PHB And PHBV Nanoparticles. Int. J. Biol. Macromol..

[33] Singh H., Balusamy S.R., Sukweenadhi J., Saravanan M., Aruchamy M., Mijakovic I., Singh P. (2025). Smart Hybrid Nanomaterials for Chronic Infections: Microbiome-Responsive and Sustainable Therapeutic Platforms. J. Nanobiotechnol..

[34] Wu Y., Ji C., Yan Z., Fang X., Wang Y., Ma Y., Li J., Jin S., Chen H., Ji S. (2025). Biological Coatings: Advanced Strategies Driving Multifunctionality and Clinical Potential in Dermal Substitutes. J. Biomed. Mater. Res. Part B Appl. Biomater..

[35] Janmohammadi M., Nazemi Z., Salehi A.O.M., Seyfoori A., John J.V., Nourbakhsh M.S., Akbari M. (2022). Cellulose-Based Composite Scaffolds for Bone Tissue Engineering and Localized Drug Delivery. Bioact. Mater..

[36] Lu P., Ruan D., Huang M., Tian M., Zhu K., Gan Z., Xiao Z. (2024). Harnessing the Potential of Hydrogels for Advanced Therapeutic Applications: Current Achievements and Future Directions. Signal Transduct. Target. Ther..

[37] Thakar H., Sebastian S.M., Mandal S., Pople A., Agarwal G., Srivastava A. (2019). Biomolecule-Conjugated Macroporous Hydrogels for Biomedical Applications. ACS Biomater. Sci. Eng..

[38] Segneanu A.E., Bejenaru L.E., Bejenaru C., Blendea A., Mogoșanu G.D., Biță A., Boia E.R. (2025). Advancements in Hydrogels: A Comprehensive Review of Natural and Synthetic Innovations for Biomedical Applications. Polymers.

[39] Engler L.G., Farias N.C., Crespo J.S., Gately N.M., Major I., Pezzoli R., Devine D.M. (2023). Designing Sustainable Polymer Blends: Tailoring Mechanical Properties and Degradation Behaviour in PHB/PLA/PCL Blends in a Seawater Environment. Polymers.

[40] Rana D., Arulkumar S., Vishwakarma A., Ramalingam M. (2015). Considerations on Designing Scaffold for Tissue Engineering. Instem Cell Biology and Tissue Engineering in Dental Sciences.

[41] Raziyan M.S., Palevicius A., Perkowski D., Urbaite S., Janusas G. (2024). Development and Evaluation of 3D-Printed PLA/PHA/PHB/HA Composite Scaffolds for Enhanced Tissue-Engineering Applications. J. Compos. Sci..

[42] Misra S., Hascall V.C., Markwald R.R., Ghatak S. (2015). Interactions between Hyaluronan and Its Receptors (CD44, RHAMM) Regulate the Activities of Inflammation and Cancer. Front. Immunol..

[43] Song J.M., Im J., Nho R.S., Han Y.H., Upadhyaya P., Kassie F. (2019). Hyaluronan-CD44/RHAMM Interaction-Dependent Cell Proliferation and Survival in Lung Cancer Cells. Mol. Carcinog..

[44] Farjaminejad S., Farjaminejad R., Hasani M., Garcia-Godoy F., Abdouss M., Marya A., Harsoputranto A., Jamilian A. (2024). Advances and Challenges in Polymer-Based Scaffolds for Bone Tissue Engineering: A Path Towards Personalized Regenerative Medicine. Polymers.

[45] Zhao Y., Wang X., Qi R., Yuan H. (2023). Recent Advances of Natural-Polymer-Based Hydrogels for Wound Antibacterial Therapeutics. Polymers.

[46] Oliveira C., Sousa D., Teixeira J.A., Ferreira-Santos P., Botelho C.M. (2023). Polymeric Biomaterials for Wound Healing. Front. Bioeng. Biotechnol..

[47] Wang L., Zhou F., Xie W. (2025). Advances in Hyaluronic Acid-Based Biomaterials: Applications in Cancer Therapy, Wound Healing, And Disease Management. J. Mater. Sci. Mater. Med..

[48] Prakash P., Lee W.H., Loo C.Y., Wong H.S., Parumasivam T. (2022). Advances in Polyhydroxyalkanoate Nanocarriers for Effective Drug Delivery: An Overview and Challenges. Nanomaterials.

[49] Niculescu A.G., Grumezescu A.M. (2021). Polymer-Based Nanosystems: A Versatile Delivery Approach. Materials.

[50] El-Sherbiny G.M., Alluqmani A.J., Elsehemy I.A., Kalaba M.H. (2024). Antibacterial, Antioxidant, Cytotoxicity, And Phytochemical Screening of Moringa Oleifera Leaves. Sci. Rep..

[51] Chiș A., Noubissi P.A., Pop O.L., Mureșan C.I., Fokam Tagne M.A., Kamgang R., Fodor A., Sitar-Tăut A.V., Cozma A., Orășan O.H. (2023). Bioactive Compounds in *Moringa oleifera*: Mechanisms of Action, Focus on Their Anti-Inflammatory Properties. Plants.

[52] Ansari S., Srivastava S., Yadav P., Parashar P. (2025). *Moringa oleifera*-Loaded Hydrogel: Assessment of Wound Healing Potential in an Animal Model. Drug Deliv. Lett..

[53] Ruttarattanamongkol K., Petrasch A. (2015). Antimicrobial activities of *Moringa oleifera* seed and seed oil residue and oxidative stability of its cold pressed oil compared with extra virgin olive oil. Songklanakarin J. Sci. Technol..

[54] Lin L., Gu Y., Cui H. (2019). Moringa Oil/Chitosan Nanoparticles Embedded Gelatin Nanofibers for Food Packaging Against Listeria Monocytogenes and Staphylococcus Aureus on Cheese. Food Packag. Shelf Life.

[55] Peñaloza J.P., Márquez-Miranda V., Cabaña-Brunod M., Reyes-Ramírez R., Llancalahuen F.M., Vilos C., Maldonado-Biermann F., Velásquez L.A., Fuentes J.A., González-Nilo F.D. (2017). Intracellular Trafficking and Cellular Uptake Mechanism of PHBV Nanoparticles for Targeted Delivery in Epithelial Cell Lines. J. Nanobiotechnol..

[56] Wang H., Zhao Y., Wu Y., Hu Y.L., Nan K., Nie G., Chen H. (2011). Enhanced Anti-Tumor Efficacy by Co-Delivery of Doxorubicin and Paclitaxel with Amphiphilic Methoxy PEG-PLGA Copolymer Nanoparticles. Biomaterials.

[57] Guo J., Song C., Lü J., Yu L., Situ W. (2022). PHA Composite Hyaluronic Acid Microspheres and Preparation Method and Uses Thereof.

[58] Pachiyappan S., Shanmuganatham Selvanantham D., Kuppa S.S., Chandrasekaran S., Samrot A.V. (2019). Surfactant-Mediated Synthesis of Polyhydroxybutyrate (PHB) Nanoparticles for Sustained Drug Delivery. IET Nanobiotechnol..

[59] Karmakar S.A. (2019). Particle Size Distribution and Zeta Potential Based on Dynamic Light Scattering: Techniques to Characterize Stability and Surface Charge Distribution of Charged Colloids. Recent Trends Mater. Phys. Chem..

[60] Roldan-Cruz C., Vernon-Carter E.J., Alvarez-Ramirez J.H. (2016). Assessing the Stability of Tween 80-Based O/W Emulsions with Cyclic Voltammetry and Electrical Impedance Spectroscopy. Colloids Surf. A Physicochem. Eng. Asp..

[61] Ardizzoni A., Neglia R.G., Baschieri M.C., Cermelli C., Caratozzolo M., Righi E., Palmieri B., Blasi E. (2011). Influence of hyaluronic acid on bacterial and fungal species, including clinically relevant opportunistic pathogens. J. Mater. Sci. Mater. Med..

[62] Lauderdale T.L., Chapin K.C., Murray P.R., Baron E.J., Pfaller M.A., Tenover F.C., Yolken R.H. (1999). Manual of Clinical Microbiology.

[63] Lin Y., Guan Y., Dong X., Ma Y., Wang X., Leng Y., Wu F., Ye J.W., Chen G.Q. (2021). Engineering *Halomonas bluephagenesis* as A Chassis for Bioproduction from Starch. Metab. Eng..

[64] Cuéllar-Gaona C.G., Ibarra Alonso M.C., Narro-Céspedes R.I., Téllez-Rosas M.M., Reyna Martínez R., Luévanos Escareño M.P. (2023). Novel Studies in The Designs of Natural, Synthetic, and Compound Hydrogels with Biomedical Applications. Rev. Mex. Ing. Biomédica.

[65] Mohammed M., Devnarain N., Elhassan E., Govender T. (2022). Exploring the applications of hyaluronic acid-based nanoparticles for diagnosis and treatment of bacterial infections. Wiley Interdiscip. Rev. Nanomed. Nanobiotechnol..

[66] Zhang Z., Li J., Ma L., Yang X., Fei B., Leung P.H., Tao X. (2020). Mechanistic Study of Synergistic Antimicrobial Effects Between Poly (3-Hydroxybutyrate) Oligomer and Polyethylene Glycol. Polymers.

[67] Shen C.F., Tremblay S., Sabourin-Poirier C., Burney E., Broussau S., Manceur A., Rodenbrock A., Voyer R., Loignon M., Ansorge S. (2022). Culture Media Selection and Feeding Strategy for High-Titer Production of a Lentiviral Vector by Stable Producer Clones Cultivated at High Cell Density. Bioprocess Biosyst. Eng..

[68] Yadav J., Balabantaray S., Patra N. (2017). Statistical Optimization of Fermentation Conditions for The Improved Production of Poly-Β-Hydroxybutyrate from *Bacillus subtilis*. Chem. Eng. Commun..

[69] Olatunji O. (2016). Natural Polymers.

[70] Yang J.E., Park S.J., Kim W.J., Kim H.J., Kim B.J., Lee H., Shin J., Lee S.Y. (2018). One-Step Fermentative Production of Aromatic Polyesters from Glucose by Metabolically Engineered *Escherichia coli* Strains. Nat. Commun..

[71] Choi S.Y., Park S.J., Kim W.J., Yang J.E., Lee H., Shin J., Lee S.Y. (2016). One-Step Fermentative Production of Poly (Lactate-Co-Glycolate) From Carbohydrates in *Escherichia coli*. Nat. Biotechnol..

[72] Sukan A., Roy I., Keshavarz T. (2015). Dual Production of Biopolymers from Bacteria. Carbohydr. Polym..

[73] Yang F., Wang H., Zhao C., Zhang L., Liu X., Park H., Yuan Y., Ye J.W., Wu Q., Chen G.Q. (2024). Metabolic Engineering of *Halomonas bluephagenesis* for The Production of Five-Carbon Molecular Chemicals Derived From L-Lysine. Metab. Eng..

[74] Park H., Toogood H.S., Chen G.Q., Scrutton N.S. (2023). Co-Production of Biofuel, Bioplastics, And Biochemicals During Extended Fermentation of *Halomonas bluephagenesis*. Microb. Biotechnol..

[75] Jiang X.R., Yan X., Yu L.P., Liu X.Y., Chen G.Q. (2021). Hyperproduction of 3-hydroxypropionate by *Halomonas bluephagenesis*. Nat. Commun..

[76] Bhattacharya S., Singh D., Aich J., Shete M.B. (2022). Development and Characterization of Hyaluronic Acid Surface Scaffolds, Encorafenib-Loaded Polymeric Nanoparticles for Colorectal Cancer Targeting. Mater. Today Commun..

[77] Ladhari S., Vu N.N., Boisvert C., Saidi A., Nguyen-Tri P. (2023). Recent Development of Polyhydroxyalkanoates (PHA)-Based Materials for Antibacterial Applications: A Review. ACS Appl. Bio Mater..

[78] Ahmed S., Keniry M., Padilla V., Anaya-Barbosa N., Javed M.N., Gilkerson R., Gomez K., Ashraf A., Narula A.S., Lozano K. (2023). Development of Pullulan/Chitosan/Salvianolic Acid Ternary Fibrous Membranes and Their Potential for Chemotherapeutic Applications. Int. J. Biol. Macromol..

[79] Ata S., Rasool A., Islam A., Bibi I., Rizwan M., Azeem M.K., Iqbal M. (2020). Loading of Cefixime to Ph-Sensitive Chitosan-Based Hydrogel and Investigation of Controlled Release Kinetics. Int. J. Biol. Macromol..

[80] Xiang J., Shen L., Hong Y. (2020). Status and Future Scope of Hydrogels in Wound Healing: Synthesis, Materials, And Evaluation. Eur. Polym. J..

[81] Kim N.G., Chandika P., Kim S.C., Won D.H., Park W.S., Choi I.W., Lee S.G., Kim Y.M., Jung W.K. (2023). Fabrication and Characterization of Ferric Ion Cross-Linked Hyaluronic Acid/Pectin-Based Injectable Hydrogel with Antibacterial Ability. Polymer.

[82] Tenje M., Cantoni F., Hernández A.M., Searle S.S., Johansson S., Barbe L., Antfolk M., Pohlit H. (2020). A Practical Guide to Microfabrication and Patterning of Hydrogels for Biomimetic Cell Culture Scaffolds. Organs-On-A-Chip.

[83] Aswathy S.H., Narendrakumar U., Manjubala I. (2020). Commercial Hydrogels for Biomedical Applications. Heliyon.

[84] Kim A.R., Lee S.L., Park S.N. (2018). Properties and In Vitro Drug Release of Ph-and Temperature-Sensitive Double Cross-Linked Interpenetrating Polymer Network Hydrogels Based on Hyaluronic Acid/Poly (N-Isopropylacrylamide) For Transdermal Delivery of Luteolin. Int. J. Biol. Macromol..

[85] Radaeva I.F., Kostina G.A., Il’ina S.G., Kostyleva R.N. (2001). Antimicrobial Activity of Hyaluronic Acid. Zhurnal Mikrobiol. Epidemiol. Immunobiol..

[86] Munir F., Safdar W., Ahmed S., Navid M.T., Ali M., Ahmed I. (2025). Production, Characterization, And Antimicrobial Activity of Polyhydroxyalkanoates Synthesized by *Bacillus* species Against Skin Pathogens. RSC Adv..

[87] Shimizu M.T., Jorge A.O., Unterkircher C.S., Fantinato V., Paula C.R. (1995). Hyaluronidase and Chondroitin Sulphatase Production by Different Species of *Candida*. J. Med. Vet. Mycol..

[88] Khalaf K.J., Hachim L.S. (2017). Effect of Hyaluronidase Enzyme, Which Produced by Staphylococcus aureus, on Killing *Klebsiella pneumoniae* in Serum. Adv. Environ. Biol..

[89] Kadhum F.M. (2023). Optimization of Hyaluronidase Enzyme Conditions Produced from *Pseudomonas aerigenosa* Isolated from Diabetic Foot Ulcer Patients. J. Med. Life Sci..

[90] Hu M., Chua S.L. (2025). Antibiotic-Resistant *Pseudomonas aeruginosa*: Current Challenges and Emerging Alternative Therapies. Microorganisms.

[91] Jack K.S., Velayudhan S., Luckman P., Trau M., Grøndahl L., Cooper-White J. (2009). The Fabrication and Characterization of Biodegradable HA/PHBV Nanoparticle–Polymer Composite Scaffolds. Acta Biomater..

[92] Sadiq S.I., Ghafil J.A. (2025). Polyhydroxybutyrate Nanoparticles Improve the Sensitivity of *Pseudomonas aeruginosa* to Ceftriaxone and Reduce the Biofilm Formation In Vitro. Polim. Med..

[93] Ghafil J.A., Al-Muffti S.A. (2023). Effect of Polyhydroxybutyrate Nano-Particles (PHB-Nps) In Mice Kidney Tissues: Histopathological Investigation. World J. Exp. Biosci..

[94] Zhang P., Lin L., Zang D., Guo X., Liu M. (2017). Designing Bioinspired Anti-Biofouling Surfaces Based on a Super-wettability Strategy. Small.

[95] Ibberson C.B., Jones C.L., Singh S., Wise M.C., Hart M.E., Zurawski D.V., Horswill A.R. (2014). *Staphylococcus aureus* Hyaluronidase Is a Cody-Regulated Virulence Factor. Infect. Immun..

[96] Korzekwa K., Sobolewski K., Wiciejowska M., Augustyniak D. (2025). Antimicrobial Activity Versus Virulence Potential of Hyaluronic Acid: Balancing Advantages and Disadvantages. Int. J. Mol. Sci..

[97] Alharbi M.S., Alshehri F.A., Alobaidi A.S., Alrowis R., Alshibani N., Niazy A.A. (2023). High Molecular Weight Hyaluronic Acid Reduces the Growth and Biofilm Formation of The Oral Pathogen *Porphyromonas gingivalis*. Saudi Dent. J..

[98] Gariboldi S., Palazzo M., Zanobbio L., Selleri S., Sommariva M., Sfondrini L., Cavicchini S., Balsari A., Rumio C. (2008). Low Molecular Weight Hyaluronic Acid Increases the Self-Defense of Skin Epithelium by Induction of Β-Defensin 2 Via TLR2 And TLR4. J. Immunol..

[99] Dubashynskaya N.V., Bokatyi A.N., Gasilova E.R., Dobrodumov A.V., Dubrovskii Y.A., Knyazeva E.S., Nashchekina Y.A., Demyanova E.V., Skorik Y.A. (2022). Hyaluronan-Colistin Conjugates: Synthesis, Characterization, And Prospects for Medical Applications. Int. J. Biol. Macromol..

