# Supplementary material for: Sustainable Production of High-Performance Antimicrobial Scaffold via an Engineered Halomonas Dual-Product Factory
Source: Biomolecules. 2026 Jun 17;16(6):889. doi: 10.3390/biom16060889 (PMC13297420; doi:10.3390/biom16060889)
Supplement: Supplementary file 1 [file biomolecules-16-00889-s001.zip › biomolecules-4317305-supplementary.pdf]

A. The Calculation Formula for PHB determination

1. Quantitative Calculation:

Gas Chromatography (GC) results can determine the mass of PHB in your vial using the peak area ratio:

$$Mass_{PHB} = \frac{Area_{sample}}{Area_{ES}} \times \frac{Mass_{ES}}{RF}$$

Where:

Area $_{sample}$: Peak area of the methyl hydroxybutyrate.
Area $_{ES}$: Peak area of the Internal Standard.
Mass $_{ES}$: The known amount of internal standard you added.
RF: The Response Factor (calculated during calibration with pure PHB).
To find the PHB content (wt%), you compare the peak area of your sample against a standard curve made with pure PHB.

The standard formula is: $PHB\ content\,(\%) = \left(\frac{Mass\ or\ f\ PHB\,(g)}{Total\ ma\ ss\ or\ f\ d\,r\,i\,e\,d\ cells\,(g)} \right) \times 100$

2. Response Factor (RF)

The Response Factor is a measure of the detector's sensitivity to a specific compound relative to a reference. In PHB analysis, the detector (usually an FID) might "react" more strongly to your internal standard (like benzoic acid) than it does to the 3-hydroxybutyrate methyl ester derived from your PHB. The equation expresses the ratio between the signal (peak area) and the actual concentration. *The equation* calculates the absolute amount of a substance directly from its own peak.

$$RF = \frac{Area_{std}}{Concentration_{std}}$$

3. Relative Response Factor / Correction Factor (CF)

The Correction Factor (of ten called the Relative Response Factor) is what you use when you have an Internal Standard (IS). Since you know exactly how much IS you added to the vial, you use the CF to "correct" the PHB peak area relative to that standard. It accounts for the fact that 1 gram of PHB and 1 gram of Internal Standard will produce different peak areas.

The equation:

$$CF = \frac{(Area_{ES} / Mass_{ES})}{(Area_{PHB} / Mass_{PHB})}$$

This is the "multiplier" you use in your final calculation to ensure that the ratio of the peaks accurately reflects the ratio of the masses.

B. GC-MS results of PHB extraction from *H. bluephagenesis* TD01 strains.

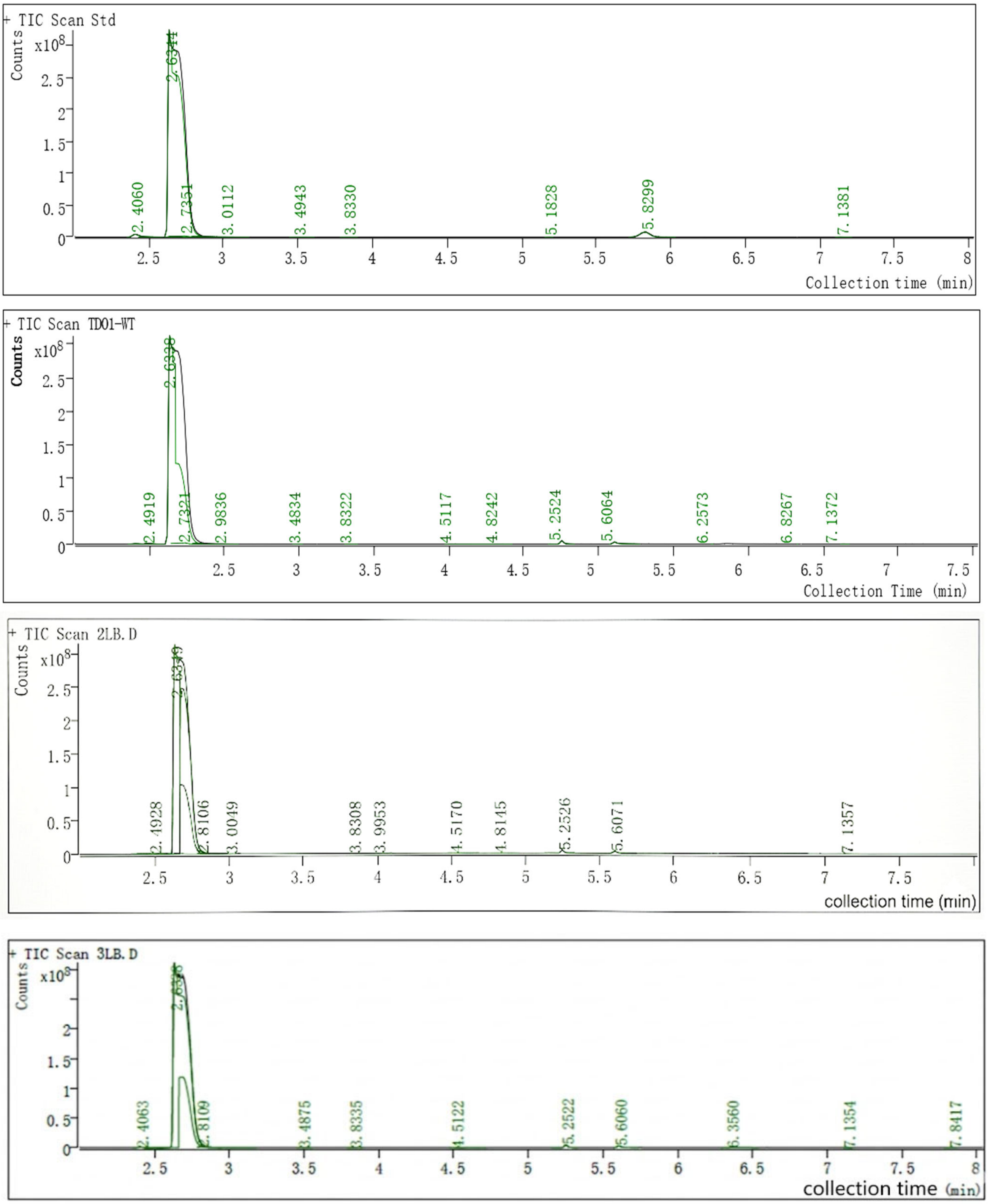

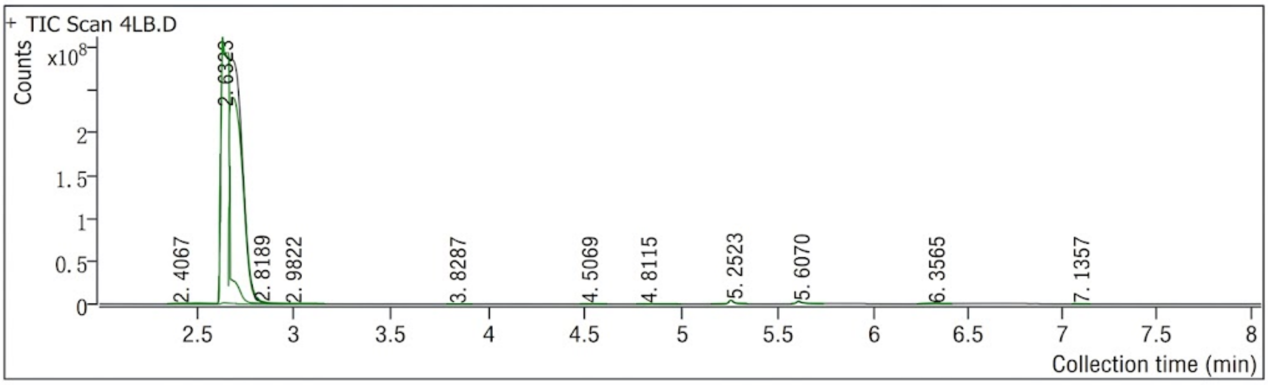

Figure S1: Mass spectra (m/z vs. counts) of cell extracts for PHB characterization by the H. bluephagenesis TD01 wild-type strain (TD01-WT), TD01-pmHAS strain (2LB), and TD01-araBAD-pmHAS strains (induced 3LB and non-induced 4LB) [1], compared against a commercial P3HB standard (Std).

References

[1] Marwan-Abdelbaset E, Lu X, Tan D, et al. Engineering Halomonas bluephagenesis TD01 as a Robust Chassis for the Sustainable Production of Hyaluronic Acid. Biomolecules 2026, 16(6), 846.
